# Demyelination and neurodegeneration early in experimental autoimmune encephalomyelitis contribute to functional deficits in the anterior visual pathway

**DOI:** 10.1038/s41598-024-73792-z

**Published:** 2024-10-14

**Authors:** Maria T. Sekyi, Micah Feri, Shane Desfor, Kelley C. Atkinson, Batis Golestany, Fernando Beltran, Seema K. Tiwari-Woodruff

**Affiliations:** Division of Biomedical Sciences, School of Medicine, University of California Riverside, Rm 3140, Multidisciplinary Research Building, 900 University Ave, Riverside, CA 92521 USA

**Keywords:** Multiple sclerosis, Optic nerve, Retinal ganglion cells, NanoString RNA, Visual evoked potentials, Optical coherence tomography, Demyelination, Axon damage, Neuroscience, Neurology

## Abstract

Impaired visual function is a prevalent feature of optic neuritis (ON) in multiple sclerosis (MS). Abnormal visual evoked potential (VEP) findings of increased latencies, reduced amplitudes and abnormal waveforms as well as decreased retinal nerve fiber layer (RNFL) assessed by optical coherence tomography (OCT) are hallmarks of ON-induced visual dysfunction. Here we utilized the experimental autoimmune encephalomyelitis (EAE) mouse model of MS to investigate the functional and pathological progression during early (before any clinical symptoms), peak (initial maximal clinical symptoms), and late (chronic disease for > 3 weeks) disease stages. Demyelination and initial stages of axon damage were observed in early EAE. Significant demyelination, inflammation, increased axon damage and impaired P1/N2 amplitudes and latencies by VEP were seen in middle and late EAE groups. A decrease in RNFL thickness by OCT was observed only during late EAE. NanoString analysis of optic nerves from late EAE indicated elevated inflammation-related genes, reduced myelin-related genes, and changes in axon degeneration-related genes. Early inflammatory demyelination and functional deficits of the visual pathway, if untreated, may lead to severe irrecoverable axon damage in EAE. These studies potentially help explain the progression of visual dysfunction during MS.

**Supplementary Information:**

The online version contains supplementary material available at 10.1038/s41598-024-73792-z.

## Introduction

Multiple sclerosis (MS) is a demyelinating, neurodegenerative, autoimmune disease of multifactorial etiology with significant motor, visual, and cognitive deficits. Currently there is no therapeutic intervention that induces remyelination and axon protection. There are no common biomarkers for MS and the basis of MS diagnosis is by determination of "lesions disseminated in space and time” by magnetic resonance imaging (MRI)^1–3^. Regarding MS diagnosis, the persistent question revolves around identifying the earliest signs of disease and determining the optimal therapeutic window to initiate treatment for maximum recovery potential before irreparable damage occurs. Understanding visual pathway function and dysfunction in MS can partially address the aforementioned question.

The visual pathway is highly myelinated and particularly susceptible to inflammatory demyelination. Thus, visual deficits due to optic neuritis (ON) are among the most prevalent symptoms in patients with MS^4,5^. Nearly 50% of patients suffering from MS experience ON which frequently precedes the more widely recognized motor symptoms of MS, such as muscle weakness, coordination problems, and difficulty with balance,^6^. ON-associated visual deficits include retinal nerve fiber layer (RNFL) thinning, loss of retinal ganglion cells (RGCs), and increased latency in visual evoked potentials (VEPs)^7–11^. Visual pathway pathology is commonly assessed in MS patients, using in vivo techniques such as MRI, optical coherence tomography (OCT), visual acuity tests, electroretinograms (ERGs), and VEPs^12–16^. Although in vivo assays of structural changes and functional deficits provide significant insights into disease progression within the visual pathway, they cannot be used to probe the cellular and molecular changes underlying macroscopic pathology. Experimental autoimmune encephalomyelitis (EAE) is a widely utilized mouse model of MS that shares key pathological features of MS, including development of inflammatory lesions, demyelination, axon pathology, and gliosis in the central nervous system (CNS)^17,18^.

Although the human and mouse visual systems differ in complexity, they share core similarities. Mice are excellent model organisms in which to study aspects of the human visual pathway and visual dysfunction^19^. Mouse RGC bodies are in the RNFL of the retina and receive information from photoreceptors. Our group and others have identified visual pathway pathology in EAE^20–23^. We showed significant demyelination, neurodegeneration, and inflammation in the EAE visual pathway using immunohistochemistry (IHC), retinal changes using OCT, and functional changes using ERG and VEP^23^. In addition, we revealed remyelination-induced partial recovery of visual function during chronic EAE with therapeutic estrogen receptor (ER) β ligand treatment after peak EAE^23^. The study highlighted the ability of an ERβ ligand to significantly remyelinate and functionally recover visual deficits as compared to vehicle treatment alone. However, a failure to remyelinate and functionally recover more than half of the axons in the optic nerve and optic tract prompted us to hypothesize that axon demyelination and axon damage in the visual pathway occurs early in EAE disease and continues in chronic disease. To test this hypothesis, we conducted a longitudinal EAE study to investigate the onset and progression of visual pathway dysfunction, employing pathological, functional, and RNA analyses. The findings from both ongoing and previously published studies underscore the importance of selecting a therapeutic window conducive to axonal recovery amidst inflammatory and demyelinating stress. This approach holds promise for improving prognosis and facilitating repair and recovery in conditions like EAE and MS.

## Results

### Persistent increase in clinical scores during early, middle and late EAE disease progression.

**Fig. 1:**
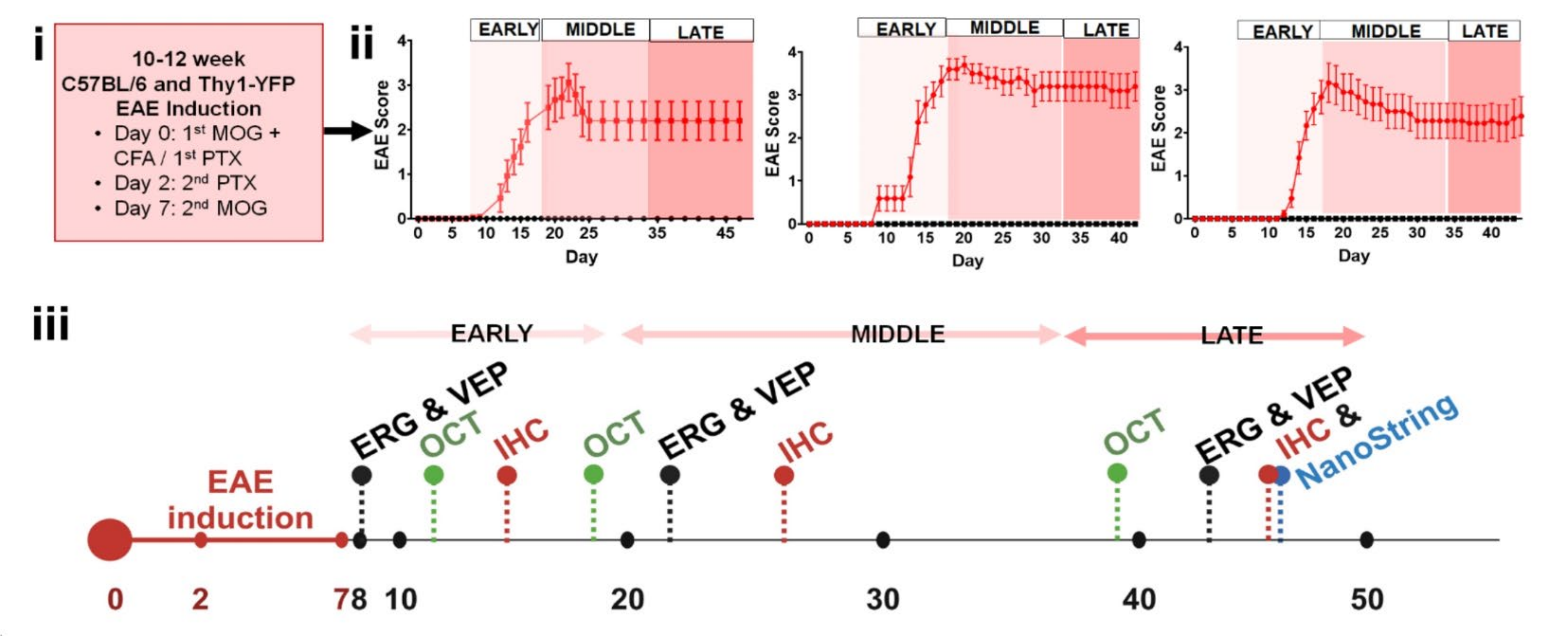
Experimental design for longitudinal assessment of visual pathway pathology. (i) Thy1-YFP and/or C57BL/6 male mice were induced with MOG_35-55_ peptide on Day 0 and Day 7. On Day 0 and Day 2, mice were injected with pertussis (PTX). (ii) Three of four representative EAE experiments are shown. EAE mice (red) displayed early signs of clinical disease symptoms. Early EAE was evaluated between days ~ 8–17. Middle EAE was evaluated between days ~ 18–35 and late EAE was evaluated between days ~ 36–47. (iii) After EAE induction, a minimum of n = 8 mice/group were subjected to OCT, electroretinography (ERG) and visual evoked potential (VEP) recordings on days 8, 21, and 42. After each timepoint n = 8 mice/group/EAE experiment were euthanized for immunohistochemistry (IHC). To assess change in mRNA transcripts during late EAE (day 45), n = 4 mice/group were euthanized, and the optic nerve was dissected for RNA extraction followed by NanoString Neuropathology Panel analysis. Figure 1iii created in BioRender. Feri, M. (2020) BioRender.com/x06w403.

Clinical signs after EAE induction were first observed between days 8–14 with peak clinical scores observed between days 18–24 which persisted into late EAE in all mice as compared to normal mice Fig. 1i). The progression and severity of EAE clinical scores are consistent with previously published results^17,18,24,25^. EAE clinical scores for three separate experiments are shown in Fig. 1 ii. OCT, ERG, and VEP were performed in post-EAE early (~ day 8–17), middle (~ day 18–35), and late (~ day 36–47) time points. A group of mice were euthanized at each time point for immunohistochemistry (IHC) analysis. Optic nerves from normal and late EAE groups were used to assess neuropathology related RNA changes.

67

### A significant RNFL decrease in late EAE is observed with OCT retinal imaging

**Fig. 2:**
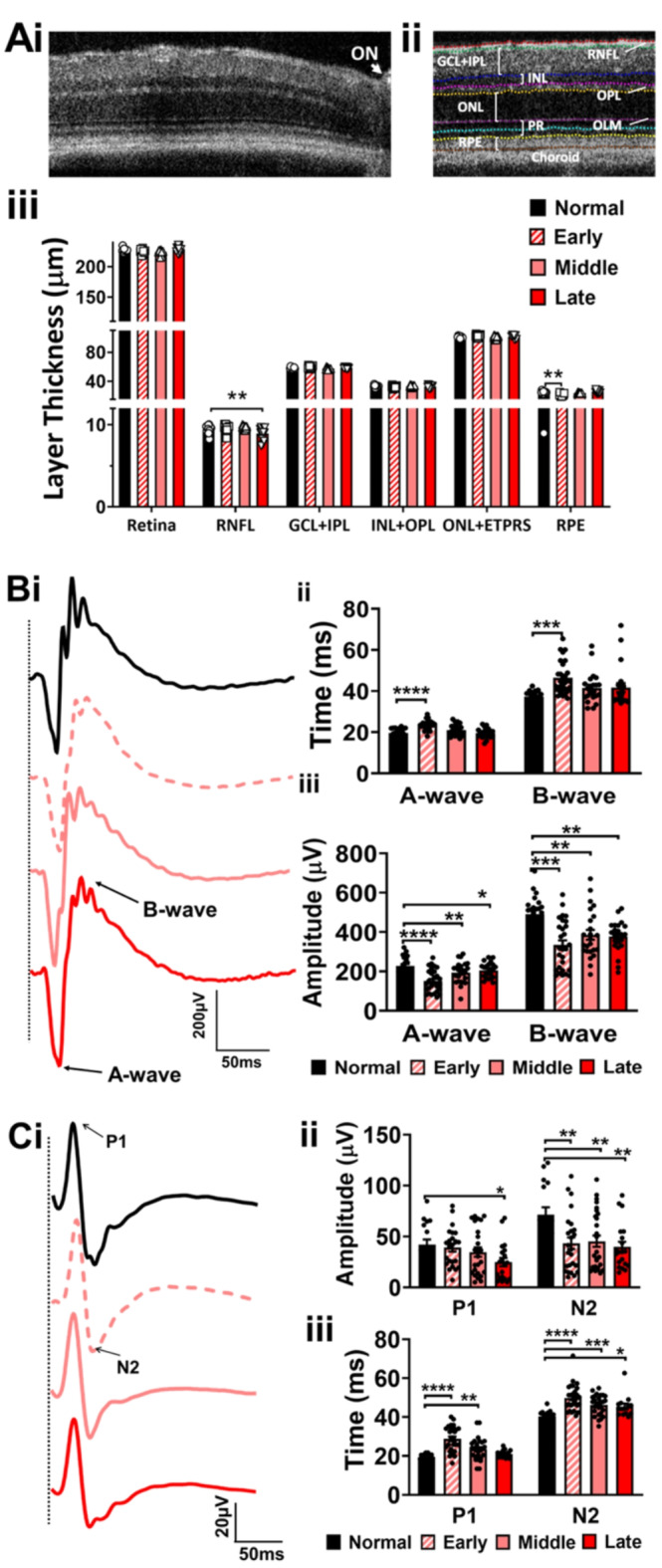
(**A**) A representative image of an OCT scan from a normal mouse with the optic nerve (ON) demarcated with an arrow is shown (**A i**). Individual layers are segmented for calculation of individual layer thicknesses for: whole retina, RNFL, PL, INL, OPL, ONL, OLM, ETPRS, RPE, and choroid (**A ii**). Quantification of layer thicknesses show no difference in whole retina, combined GCL + OPL, combined INL + OPL, or combined ONL + ETPRS. Early EAE mice show decreased RPE thickness compared to normal. Only late EAE mice showed a decrease in the RNFL thickness as compared to normal (**A iii**). (**B**) ERG latencies are impaired early in EAE, whereas amplitude deficits are evident throughout disease. Retinal function was assessed in normal, early EAE, middle EAE, and late EAE mice. For ERG recordings, silver thread electrodes were placed on the cornea and reference electrodes in the snout. (**B i**) Representative ERG responses from all treatment groups are shown with A and B wave components demarcated with black arrows on the late EAE waveform (red). (**B ii**) Early EAE mice showed an increase in ERG latency compared to normal. Middle and late EAE mice show no difference in latency. (**B ii**) A decrease in ERG amplitude was observed throughout all timepoints of disease. (**C**) Visual function was assessed from normal, early EAE, middle EAE, and late EAE mice. (**C i**) Representative VEP responses from all treatment groups are shown with P1 and N2 components defined with black arrows on the late EAE waveform (red). (**C ii**) Early and middle EAE mice show no changes in P1 amplitude compared to normal. Only late EAE mice show a decrease in VEP N2 amplitude. This decrease in N2 amplitude continued during middle and late EAE. (**C iii**) An increase in P1 and N2 latency was observed in early EAE mice. This increase in latency continued throughout the rest of the timepoints. n = 5–8 mice/group. All graphs represent mean + SEM. *p<0.05, **P<0.01, ***p<0.001, ****p<0.0001 level.

The thinning of the peripapillary RNFL, ganglion cell layer (GCL), and inner plexiform layer (IPL) have been recognized as indicative of axonal degeneration in MS^26,27^ and EAE^23,28^. The temporal retina of normal and EAE mice was longitudinally imaged by OCT, approximately 0.3 mm lateral to the optic nerve (Fig. 2A i). Different layers of the retina (RNFL, GCL, IPL, inner nerve layer (INL), outer plexiform layer (OPL), outer nuclear layer (ONL), photoreceptor end tips (ETPRS), and retinal pigment epithelium (RPE)) were demarcated using an unbiased and automatic segmentation of OCT images by system software. Respective thicknesses of the demarcated layers were measured and plotted: whole retina (RNFL to RPE), RNFL (INFL to ONFL), combined GCL and IPL, retina interneuron layers (IPL + INL), photoreceptor layers (ONL + ETPRS), and RPE. Some layers were combined due to demarcating difficulty using OCT^29,30^.

Whole retina, GCL + IPL, INL to ONL, and OPL to PRS measurements did not show significant differences between normal and EAE groups (Fig. 2A ii). Only the late EAE group showed a significant decrease in the thickness of the RNFL as compared to the normal group (F (3, 52) = 6.891, p = 0.0140). The RPE layer had a significant decrease in the early (F (3, 48) = 14.21, p < 0.0001) EAE group as compared to the normal group (Fig. 2A iii).

### ERG and VEP deficits are observed in early, middle, and late EAE

ERG is an objective measure of retinal function and arises from currents generated by retinal neurons with contributions from retinal glia. It serves as a well-established non-invasive technique for assessing retinal function^31^. ERGs were performed in live, anesthetized, and dark-adapted normal, early, middle, and late EAE mice. Normal mice exhibited robust ERGs (Fig. 2B i) with average latencies of response to light stimulus of 20 ms and 40 ms and average amplitudes of 200 µV and 500 µV for A-wave and B-wave respectively (Fig. 2B ii-iii). Early EAE groups exhibited an increased latency of response after light stimulus compared to normal controls in the A-wave (F (3, 89) = 14.32, p < 0.0001) and B-wave (F (3, 89) = 6.031, p = 0.0002), which resolved during middle and late EAE (Fig. 2B i-ii). There was a significant decrease in A-wave (F (3, 88) = 12.29, p < 0.0001) and B-wave (F (3, 92) = 8.472, p < 0.0001) amplitude in early EAE that persisted through middle (A-wave ; F (3, 88) = 12.29, p = 0.0055, B-wave; (F (3, 92) = 8.472, p < 0.0054)) and late EAE disease (A-wave ; t = 2.216, df = 40, p = 0.0324, B-wave; (F (3, 92) = 8.472, p = 0.0028) (Fig. 2B i-iii).

### Afferent visual function is significantly impaired in EAE mice

VEPs represent the expression of electrical activity of the visual pathways and depend on the integrity of the visual pathway including the eye, optic nerve, optic chiasm, optic tract, optic radiation, and cerebral cortex. VEPs were recorded in early, middle, and late EAE mice and compared to normal mice using published protocols^23^ (Fig. 2C). Normal groups exhibited robust VEPs consisting of an initial positive P1 deflection, followed by a negative deflection, N2, and gradual return to baseline (Fig. 2C i). P1 and N2 peak amplitudes from normal groups averaged 40 µV and 60 µV, and response times measured at 20 ms and 40 ms respectively (Fig. 2C i-iii). A significant decrease in P1 amplitudes were observed during late EAE as compared to normal groups (t = 2.688, df = 35, p = 0.0362). A significant decrease in P1 latency was observed in early (F (3, 85) = 13.00, p < 0.0001) and middle (F (3, 85) = 13.00, p = 0.0010) EAE, which resolved during late EAE. Interestingly, N2 latency (F (3,83) = 13.00, early: p < 0.0001, middle: p = 0.0002, late: p = 0.0144) and N2 amplitudes (F (3,84) = 4.779, early: p = 0.0055, middle: p = 0.0093, late: p = 0.0026) were reduced at all EAE timepoints compared to normal (Fig. 2C ii-iii).

### All stages of EAE show loss of retinal ganglion cells and activation of astrocytes

**Fig. 3:**
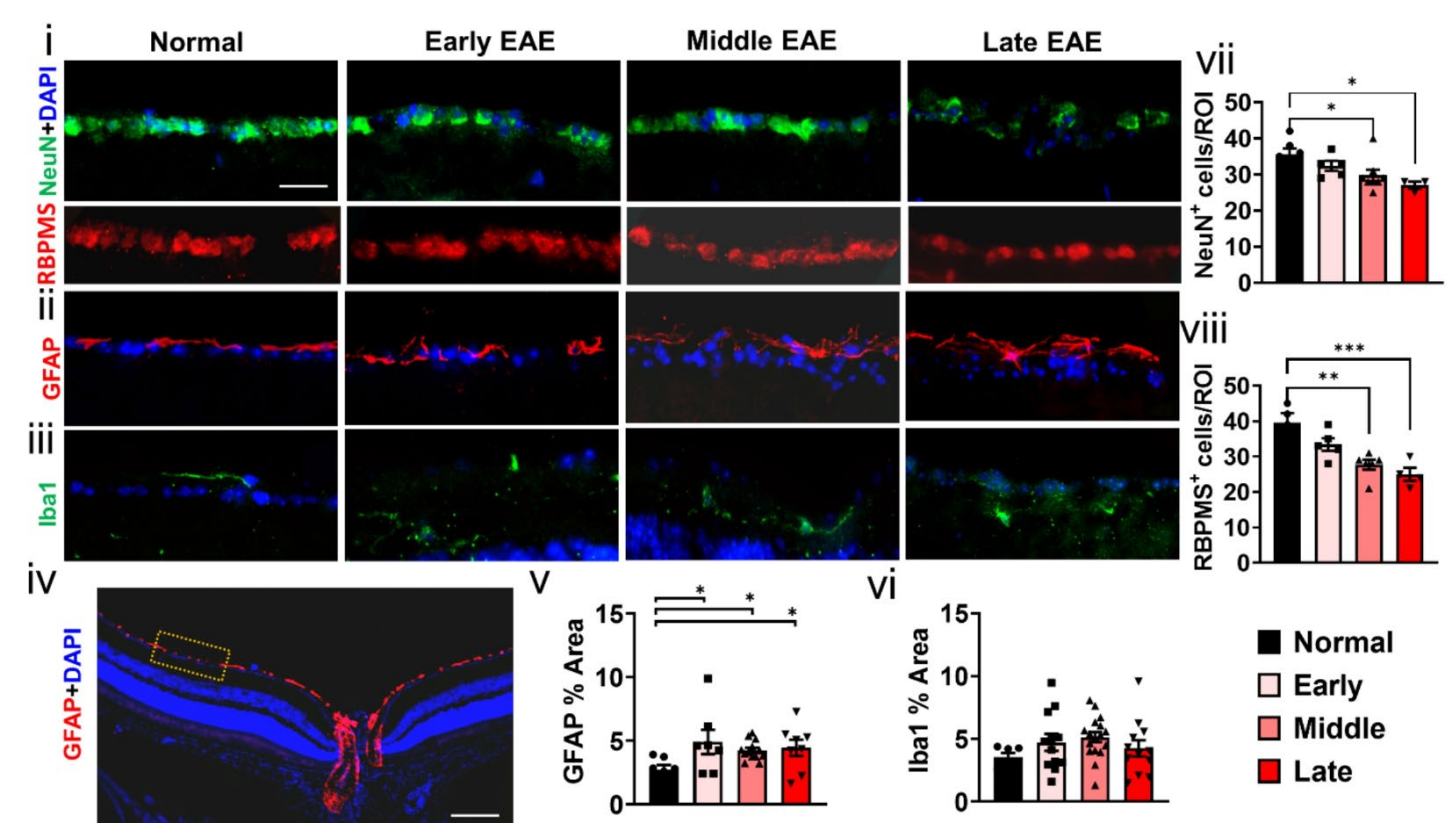
The status of RGC during EAE progression was investigated by immunostaining retinal sections with neuronal nuclear protein (NeuN; green), RBPMS (red) and DAPI (blue) (**i**). To assess the status of astrocytes (**ii**) and microglia (**iii**), IHC with GFAP (red) and Iba1 (green) antibody was used. A representative 10 × image of normal retina stained with GFAP and DAPI (blue) showing the region indicated with a dashed rectangle that was imaged for analysis. (**iv**). Quantification of GFAP immunofluorescence shows revealed activation at all EAE timepoints as compared to normal retina. (**v**). Quantification of Iba1 staining did not reveal any changes in microglia. (**vi**). A decrease in NeuN^+^ RGCs was observed in the middle and late EAE groups. (**vii**). Quantification of RBPMS^+^ RGCs showed a reduction during middle and late EAE groups as compared to normal (**viii**). At least n = 5 mice/group/EAE experiment were used. All graphs represent mean + SEM. *p < 0.05, **p < 0.01, ***p < 0.001, using ordinary one-way ANOVA with Bonferroni’s multiple comparisons test.

Our present and published results using OCT demonstrate significant decreases in the RNFL layer in the late EAE groups as compared to normal groups (Fig. 2A)^23^. Retinal atrophy, loss of RGCs, and extensive glial fibrillary acidic protein (GFAP) immunoreactivity has been observed in MS and EAE^20,23,32,33^. Neuronal nuclear protein (NeuN), a marker for post-mitotic neurons, and a RGC selective marker, RNA-binding protein with multiple splicing (RBPMS) was used to quantify RGCs (Fig. 3 i). During middle EAE, a significant reduction in NeuN^+^ cells (F (3, 23) = 6.580, p = 0.0048) was observed and continued throughout (F (3, 18) = 4.024, p = 0.0374) late EAE disease (F (3, 18) = 4.024, p = 0.0179) (Fig. 3 i, vii). RBPMS^+^ RGCS also showed a significant decrease during middle and late EAE compared to normal (F (3, 15) = 10.18, middle: p = 0.0014, late: p = 0.0005) (Fig. 3 i, viii). MOG_35-55_ peptide induced EAE is accompanied by increased astrogliosis and inflammation in the CNS^17^. To assess astrogliosis and microglia activation, retina sections were immunostained with astrocyte marker, GFAP, and microglia marker, ionized calcium binding adaptor molecule 1 (Iba1) respectively. Compared to normal sections, extensive astrocyte activation (Fig. 3 ii, iv, v) (F (3, 31) = 2.596, early: p = 0.0136, middle: p = 0.0472, late: p = 0.0416), but minimal microglia activation (Fig. 3 iii, vi) was observed in early, middle, and late EAE retinas.

### Extensive demyelination, inflammation, and axon degeneration in the EAE optic nerve

**Fig. 4:**
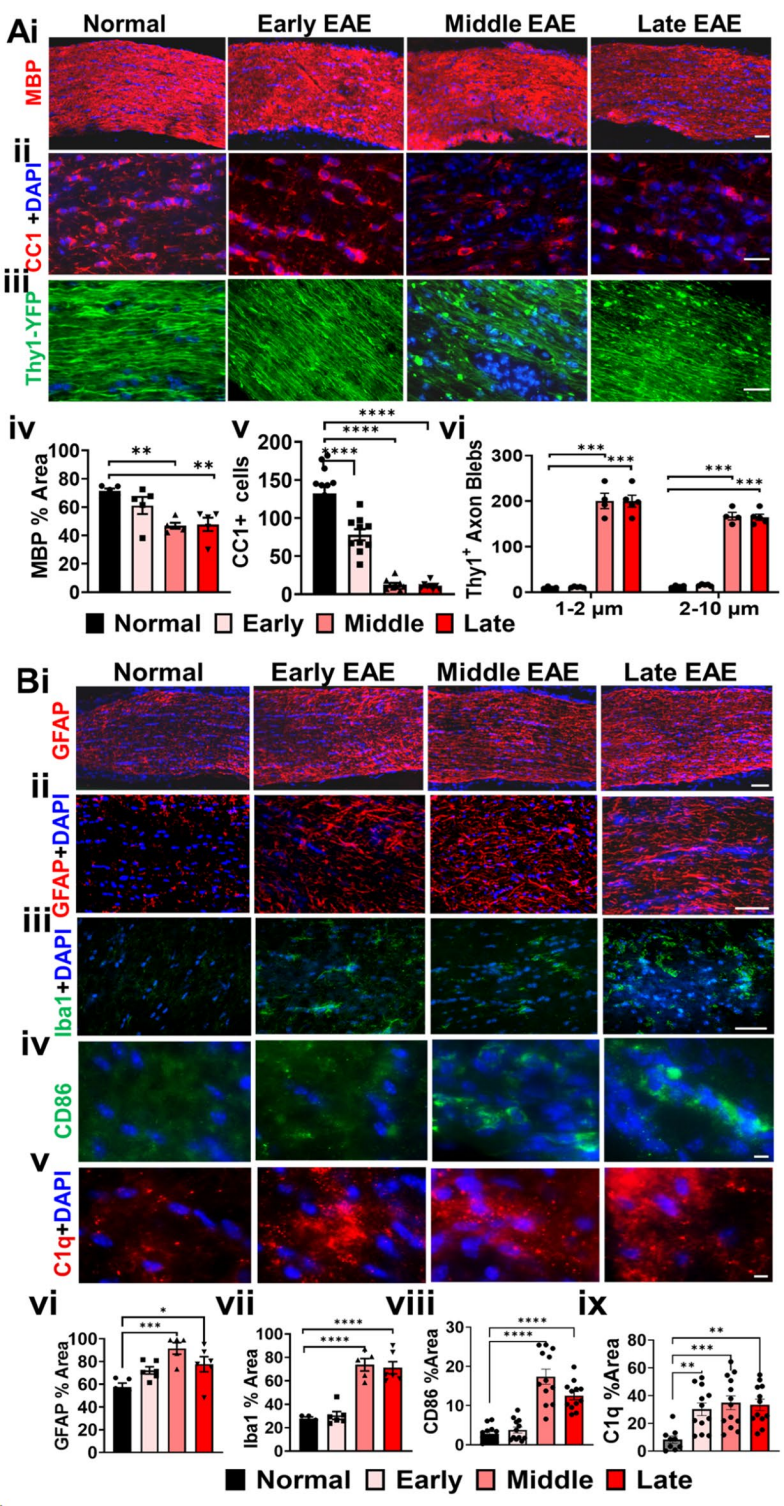
Significant demyelination, inflammation, and axon damage is present in EAE optic nerves. (**A i**) Representative images of longitudinal optic nerves are shown for normal, early, middle, and late EAE groups. To assess the extent of demyelination and axon damage during different timepoints during disease, optic nerve sections were immunostained for MBP (red) **A i**, and a marker for mature OLs, adenomatous polyposis coli(CC1; red) **A ii**. Thy1-YFP mice were used to assess the integrity of axons (green) **A iii**. A decrease in MBP staining was observed in middle EAE and persisted until late EAE compared to normal (**A iv**). A decrease in mature OLs was observed in all EAE timepoints (**A v**). Axon blebs were divided into two sizes, small blebs that are 1–2 µm and larger blebs that fall between 2–10 µm (**A vi**). Middle and late EAE had an increase in small and large axon blebs. (**A vi)**. Scale bar for **A ii** = 100 µm and **Aii, iii** = 10 µm. To assess the extent of inflammation, optic nerve sections were stained with GFAP; (red), Iba1; (green), CD86; a complex immune cell marker expressed on antigen-presenting cells, such as dendritic cells, Langerhans cells, macrophages, and B-cells (green), and C1q, a component of the complement system (red) (**B ii**). GFAP+ reactive astrocytes were enhanced in middle and late EAE (**B I, ii, vi**) Iba1 + , and CD86 + cells were also increased in middle and late EAE (**B iii, iv, vii, viii**). On the other hand, C1q reactivity was seen in all stages of EAE as compared to normal group (**B v, ix**). n = 5–8 mice/group. Scale bar for **B i-iii** = 100 µm and **B iv, v** = 10 µm. All graphs represent mean + SEM. *p<0.05, **p<0.01, ***p<0.001, ****p<0.0001 level.

CNS white matter tracts including optic nerves of EAE animals show extensive demyelination and axon damage along with inflammation^17,23,34,35^. To determine the onset of demyelination, axon damage, and inflammation in the optic nerve during EAE, IHC was performed in longitudinal optic nerve sections from early, middle, and late EAE disease (Fig. 4). To evaluate axon myelination, an antibody against myelin basic protein (MBP) was used to quantify changes in staining intensity. Optic nerves from the normal group depicted robust MBP staining (Fig. 4A i). In early EAE optic nerves, MBP levels similar to normals were observed. A significant decrease in MBP^+^ intensity was observed during middle and late EAE (F (3, 16) = 8.365, middle: p = 0.0017, late: p = 0.0024) (Fig. 4A i, iv). The decrease in MBP intensity in middle and late EAE could be due to a decrease in myelinating mature oligodendrocytes (OLs). OLs were identified by using a mature OL marker, adenomatous polyposis coli (CC1). Optic nerve sections from normal mice showed elevated levels of CC1 staining (Fig. 4A ii). Relative to normal groups, a significant decrease in CC1^+^ OL numbers (F (3, 35) = 63.67, p < 0.0001) were observed in early, middle, and late EAE groups (Fig. 4A ii, v).

It has been well established that inflammation and demyelination is predominantly accompanied by axon damage during MS and EAE^36–38^. To assess EAE-induced changes in optic nerve axons, optic nerve longitudinal sections from Thy1-YFP mice were imaged. Optic nerve sections from normal and early EAE mice revealed coherent YFP^+^ intensity in linear axons (Fig. 4A iii). However, as disease progressed to middle and late EAE, a significant decrease in Thy1‐YFP fluorescence along the axons, with significant numbers of punctate, fragmented “axonal blebs” that were 1–2 µm in size (F (3, 9) = 48.02, middle: p < 0.0001, late: p < 0.0001) and 2–10 µm (F (3, 9) = 130, middle: p < 0.0001, late: p < 0.0001) were observed (Fig. 4A iii, vi) .

To assess inflammation, optic nerves were immunostained for Iba1, GFAP, CD86, (a marker for macrophages), and C1q (a component of the complement system) (Fig. 4B). Minimal GFAP and Iba1 staining was observed in normal and early EAE optic nerve sections. During middle and late EAE, increased GFAP (F (3, 18) = 7.504, middle: p = 0.0005, late: p = 0.0252) and Iba1 (F (3, 17) = 31.66, middle: p = 0.0001, late: p = 0.0001) immunoreactivity was observed compared to the normal group (Fig. 4B i-iii, v-vii). Minimal CD86 immunoreactivity was observed in normal and early EAE optic nerves. A significant increase in CD86^+^ cells was seen during middle EAE (F (3, 44) = 34.49, p < 0.0001) and late EAE (F (3, 44) = 34.49, p = 0.0327) compared to the normal group (Fig. 4B iv, viii). Increased C1q expression was seen in early to late EAE optic nerve sections as compared to normal (F (3, 42) = 6.797, early: p = 0.0093, middle = 0.0010, late: p = 0.0023) (Fig. 4B v, ix).

### EAE induces early demyelination and OL loss in the optic tract

**Fig. 5:**
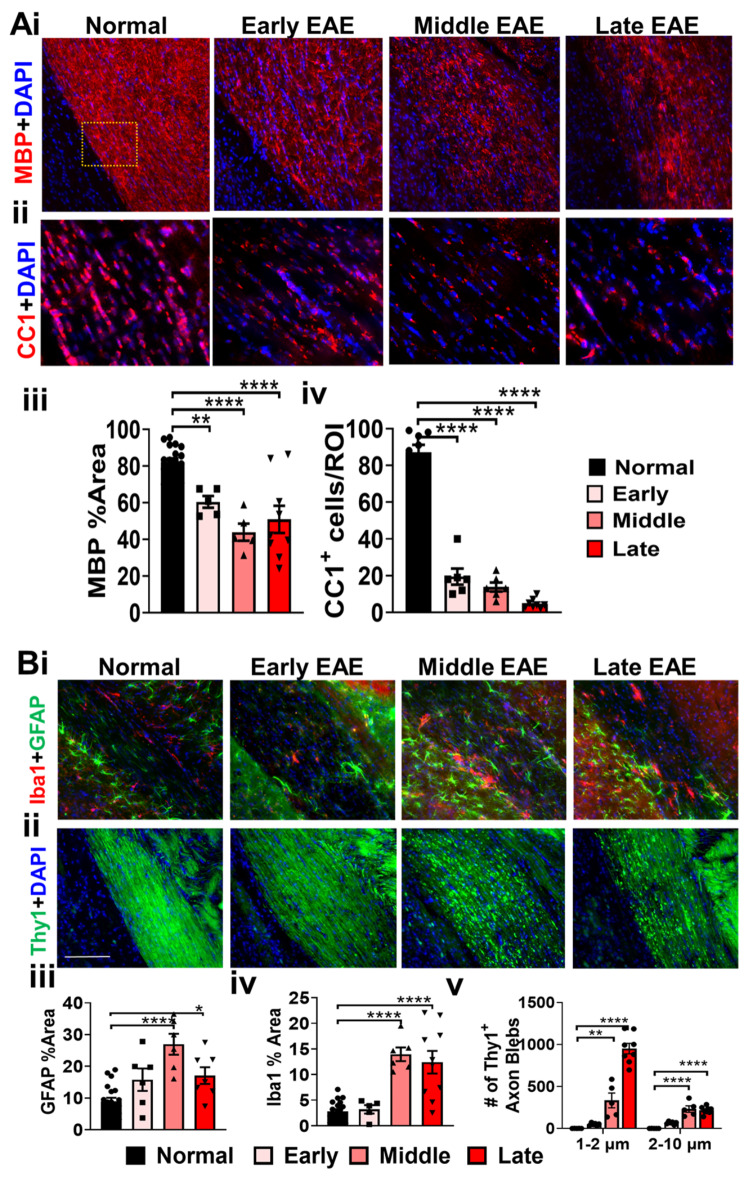
Brain sections containing the optic tract were used to assess the status of myelin and mature OLs (**A**); as well as astrocytes, microglia and changes to axons (**B**). MBP (red) density and CC1^+^ (red) OL numbers showed a significant decrease in all stages of EAE as compared to the normal group (**A i, ii, iii, iv**). Iba1 (red) and GFAP (green) reactivity was increased in middle and late EAE (**B i, iii, iv**). Axon changes were measured by assessing fragmentation and blebs. These were divided into two sizes, small blebs that are 1–2 µm and larger blebs that fall between 2–10 µm. Axon blebbing was apparent in middle and late EAE with increased axon blebs of 1–2 µm and 2–10 µm sizes. n = 5–8 mice/group. All graphs represent mean + SEM. *p < 0.05, **p < 0.01, ***p < 0.001, ****p < 0.0001 using ordinary one-way ANOVA with Bonferroni’s multiple comparisons test.

The optic tracts extend from the optic chiasm to the lateral LGN. Diffusion tensor imaging of the optic tracts in MS show association with retinal thinning and visual disability and may provide the role of the anterior visual pathway in visual dysfunction in MS and EAE^39^. Similar to the optic nerve, optic tract axons are heavily myelinated and are susceptible to EAE-induced demyelination and OL death. To characterize EAE-induced effects on myelin and OL loss, coronal sections were immunostained with anti-MBP and anti-CC1 antibodies. Normal sections showed robust MBP staining in the optic tract and adjacent hypothalamus, but minimal immunoreactivity was observed in the adjacent amygdala (Fig. 5A i, iii). Sections from EAE mice showed a significant decrease in myelin intensity in the optic tract at all EAE timepoints (F (3,33) = 16.92, early: p = 0.0098, middle: p < 0.0001, late: p < 0.0001). Significantly fewer immunoreactive CC1^+^ OLs in the optic tract was identified in all EAE timepoints as compared to normal mice (F (3,30) = 126.5, early: p < 0.0001, middle: p < 0.0001, late: p < 0.0001) (Fig. 5A ii, iv).

### Inflammation, astrogliosis and axonal damage are most prominent during peak and chronic disease stages in the optic tracts of EAE mice

EAE-induced microglial activation, astrogliosis and axonal damage in the optic tract were investigated in Thy1-YFP coronal brain sections. Sections were immunostained for Iba1 and GFAP. A small number of resident microglia and astrocytes were present in the optic tract of the normal group (Fig. 5B). Significant increases in GFAP immunoreactivity were observed in middle and late EAE optic tracts ((F (3,35) = 12.85, early: p = 0.03, middle: p < 0.0001, late: p = 0.0201) (Fig. 5B i, iii). Iba1 + microglia intensity was significantly increased in middle and late EAE as compared to normal groups (F (3,37) = 21.44, middle: p < 0.0001, late: p < 0.0001) (Fig. 5B i, iv).

To assess if axon damage discovered in the optic nerve extended to the optic tract, the optic tract of Thy1-YFP EAE animals was investigated. Coherent linear Thy1-YFP fluorescence was observed in the normal optic tract. By middle and late EAE, the optic tract axons showed fragmentation, axon swellings, and discontinuous Thy1-YFP staining with axon blebs that were 1–2 µm (F (3, 14) = 108.8, middle: p < 0.001, late: p < 0.0001) and 2–10 µm (F (3, 14) = 269.8, middle: p < 0.0001, late: p < 0.0001) in size (Fig. 5B ii, v).

### Progressive demyelination is coupled with severe OL loss in the dLGN of EAE mice

**Fig. 6:**
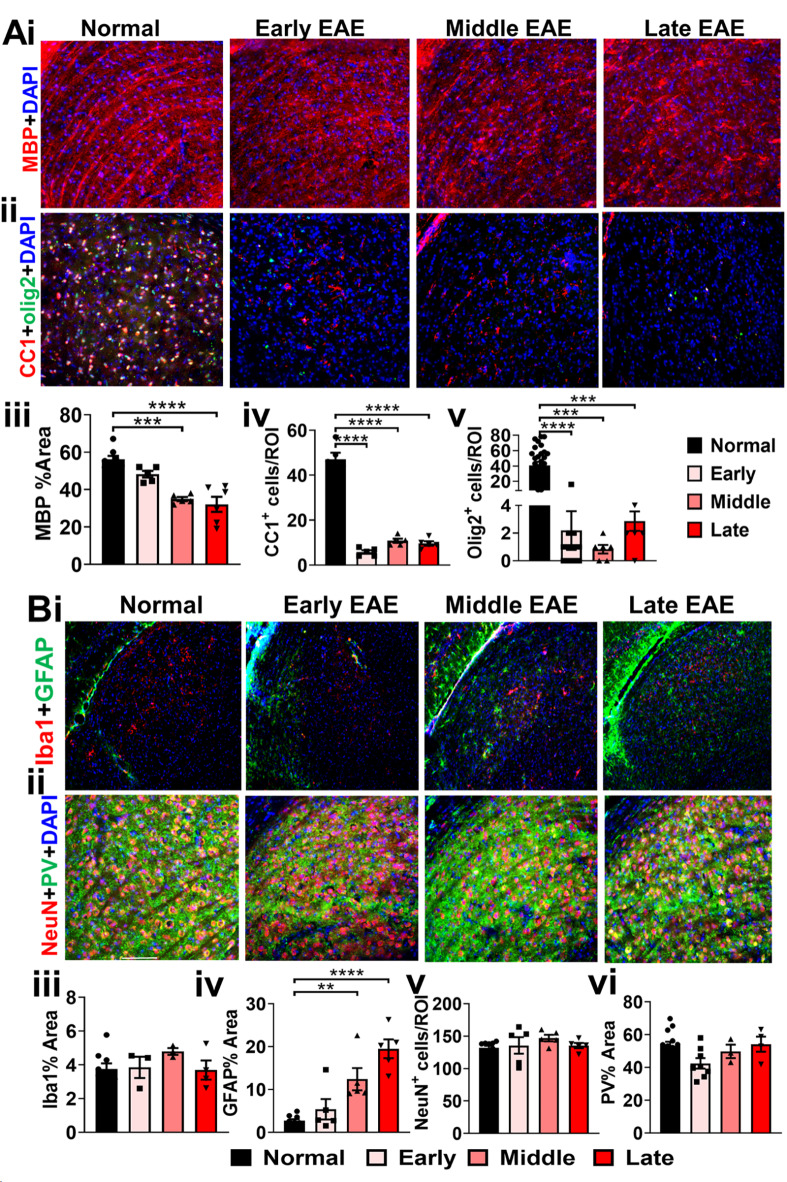
Brain sections containing the dLGN were used to assess the status of myelin and OLs (**A**); as well as astrocytes, microglia and neuronal population (**B**). MBP (red) density showed a significant decrease in middle and late EAE timepoints as compared to normal group (**A i, iii**). and CC1 + (red) OL numbers were drastically decreased in all stages of EAE (**A ii, iiv**). Iba1 (red) was unchanged, but GFAP (green) reactivity was increased in middle and late EAE (B i, iii, iv). NeuN (red) and PV (green) were similar in numbers in all groups. n = 5–8 mice/group. All graphs represent mean + SEM. *p < 0.05, **p < 0.01, ***p < 0.001, ****p < 0.0001 using ordinary one-way ANOVA with Bonferroni’s multiple comparisons test.

The dLGN not only serves as an important intermediary between the retina and the visual cortex in the afferent visual pathway, but also is significantly involved in processing and modulation of the visual signal^40,41^. The human LGN consists of six cellular layers separated by thin sheets of myelinated fibers, and thus is susceptible to MS-induced demyelination^42,43^. Loss of synaptic input from RGCs has been shown to cause significant neuronal apoptosis in the LGN. The presence of significant EAE-induced damage in RGC axons of the optic tract suggested possible downstream neuronal loss in the dLGN. Significant inflammation, demyelination, and neuronal loss in the dLGN have been observed in chronic EAE^20,23^. Here, myelin intensity (MBP) and OL (OL transcription factor 2, (olig2) and CC1)) numbers were investigated in brain sections containing the dLGN from early, middle, and late EAE and compared to normal group (Fig. 6A). The dLGN from the normal group showed highly myelinated fiber tracts interleaved between partially myelinated cellular layers (Fig. 6A). There is a decrease in a myelination of the dLGN during middle EAE (F (3,15) = 16.87, p = 0.0009) and late EAE (F (3,15) = 16.87, p < 0.0001) (Fig. 6A i, iii). The normal dLGN revealed uniform levels of olig2^+^ and CC1^+^ OLs. (Fig. 6A ii, iv, v). In contrast, all EAE timepoints showed very few olig2^+^ (F (3,43) = 14.42, early: p < 0.0001, middle: p = 0.0058, late: p = 0.0007) and CC1^+^ cells (F (3,12) = 93.51, early: p = 0.0001, middle: p = 0.0001, late: p = 0.0001) as compared to the normal dLGN (Fig. 6A ii, iv, v).

### The dLGN exhibits progressive astrogliosis in EAE, but no change in microglia, NeuN^+^ neurons, or Parvalbumin (PV) immunoreactivity

A decrease in LGN volume has been identified in MS patients with optic neuritis^43^. This decrease could be due to demyelination, inflammation and/or neuronal loss. Past studies have shown demyelination and inflammation in the LGN of EAE mice. Inflammation, astrogliosis and neuronal numbers were assessed by IHC (Fig. 6B). Minimal Iba1^+^ microglia immunoreactivity was observed in the normal LGN. (Fig. 6B i. iii). Surprisingly, no change in microglia number or morphology was observed in the LGN of early, middle, and late EAE groups (Fig. 6B i. iii). Similar GFAP^+^ immunoreactivity was seen in the normal and early EAE groups (Fig. 6B i. iv). However, by middle and late EAE, there was a significant increase in astrocyte activation (F (3,21) = 21.02, middle: p = 0.009, late: p < 0.0001).

The dLGN is comprised of a number of thalamocortical projection neurons as well as inhibitory interneurons^44^. To quantify changes in the dLGN neuronal population, brain sections with the dLGN were immunostained for NeuN and PV protein that is highly expressed in inhibitory interneurons (Fig. 6B ii, v, vi). In normal sections, NeuN staining was evident in neuronal cell nuclei, while PV staining was primarily observed in dendritic fibers and some cell bodies (Fig. 6B ii, v, vi). Interestingly, thin streaks absent of PV immunoreactivity were evident across the LGN and corresponded to the myelinated fiber tracts observed in (Fig. 6A i). None of the EAE timepoints showed differences in NeuN staining, or NeuN^+^ cell numbers. Similarly, PV immunoreactivity was also similar in normal and EAE groups, however some loss of organization was observed in sections from all timepoints (Fig. 6B ii).

### Progressive loss of myelin and mature OLs is observed in the EAE visual cortex

Axons of the LGN neurons merge into the optic radiations and project to layer VI of the visual cortex, where synaptic inputs are processed and transferred to layer II/III, V, and VI pyramidal neurons. These neurons, in conjunction with inhibitory circuits generated by cortical inhibitory neurons and feedback from higher order visual areas, form the final visual image. Although the cortex is accepted to be a gray matter region, many cortical pyramidal neurons and PV^+^ interneurons are myelinated^45–47^. Not much information is available on specifically the visual cortex pathology during EAE.

**Fig. 7:**
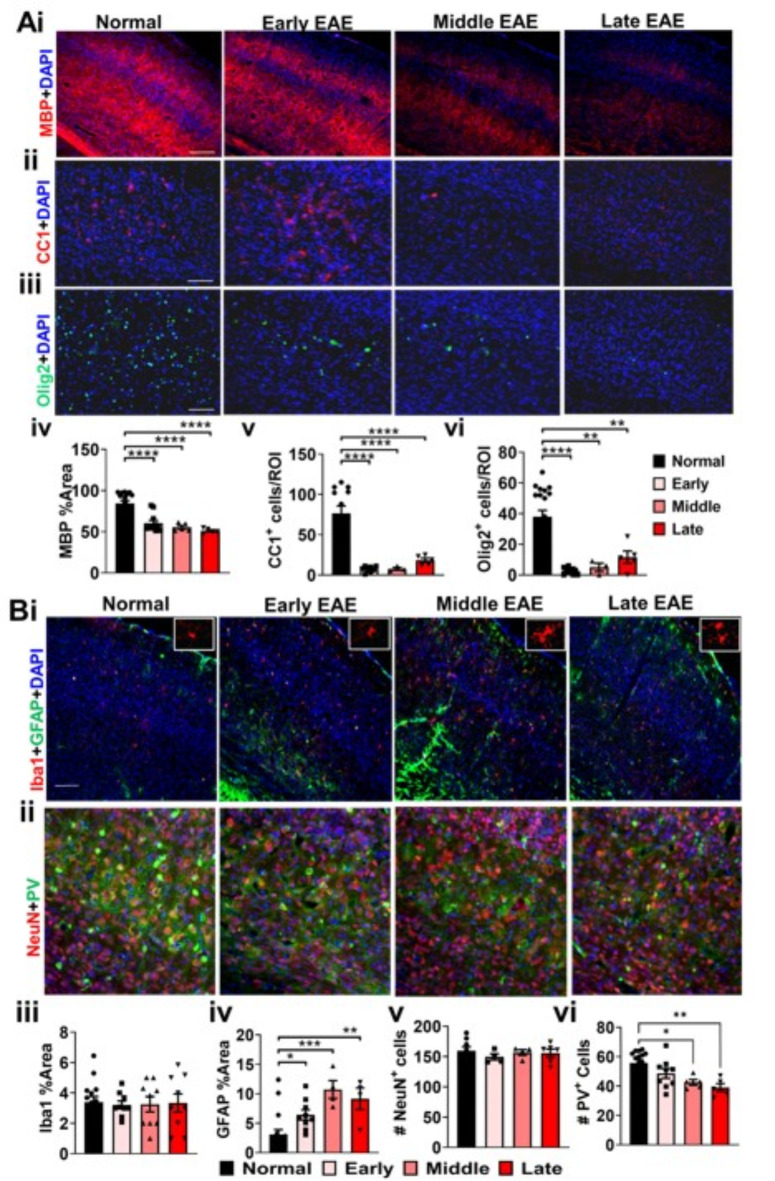
Brain sections containing the visual cortex were used to assess the status of myelin and OLs (**A**); as well as astrocytes, microglia and neuronal population (**B**). MBP (red) density showed a significant decrease in middle and late EAE timepoints as compared to normal group (**A i, iv**). and CC1^+^ (red) and olig2 (green) OLs were decreased in all stages of EAE (**A ii, iii, v, vi**). Iba1 (red) overall intensity was unchanged, however, microglia in the middle and late EAE showed hypertrophic appearance (high magnification image Iba1^+^ cells). GFAP (green) reactivity was increased in middle and late EAE (**B i, iii, iv**). Number of NeuN^+^ (red) cells were unchanged in all groups as compared to the normal groups (**B ii, v**). Interestingly, number of PV^+^ neurons were significantly decreased in middle and late EAE timepoints (**B ii, vi**). n = 5–8 mice/group. All graphs represent mean + SEM. *p < 0.05, **p < 0.01, ***p < 0.001, ****p < 0.0001 using ordinary one-way ANOVA with Bonferroni’s multiple comparisons test.

To evaluate the status of myelination and OLs in the visual cortex during EAE, layer IVA-containing brain sections were immunostained for MBP, CC1 and olig2. The visual cortex IV sections revealed robust MBP intensity (Fig. 7A). A significant decrease in myelination was observed in early, middle, and late EAE timepoints (F (3,33) = 18.21, early: p = 0.0001, middle: p = 0.0001, late: p = 0.0001) (Fig. 7A i, iv). Similarly, there was a significant decrease in olig2^+^ (F(3,33) = 16.77, early: p = 0.0001, middle: p = 0.0020, late: p = 0.0022) and CC1^+^ (F(3,26) = 23.48, early: p = 0.0001, middle: p = 0.0001, late: p = 0.0001) OLs in early, middle, and late EAE groups compared to those seen in the normal visual cortex group (Fig. 7A ii, iii, v, vi).

### Astrogliosis, and a progressive loss of PV^+^ interneurons during EAE progression

Cortical microglia have been shown to become ramified and hypertrophied in chronic stages of EAE^17^. Astrocytes in the cortex have been shown to express higher levels of GFAP, indicative of gliotic responses to injury at peak, remitting, and late disease stages of the relapsing remitting EAE model and at peak disease in the MOG_35-55_ peptide EAE model^48,49^. To evaluate cortical changes in microglial and astrocytic reactivity in V1, coronal sections containing V1 were immunostained for Iba1 and GFAP, respectively. Overall Iba1 immunoreactivity density during EAE did not change as compared to normal groups, however, the cells looked more ramified in middle and late EAE (Fig. 7B i, iii). EAE groups showed a significant increase in GFAP + immunoreactivity (F (3,30) = 8.805, early: p = 0.0497, middle: p = 0.0005, and late: p = 0.00054) as compared to the normal group (Fig. 7B i, iv).

Layer V1 of the visual cortex undergoes atrophy in MS, indicating the possibility of V1 neuronal death^7,8,50^. To quantify EAE-induced neuronal pathology in V1, coronal sections from normal and EAE mice were immunostained for NeuN and PV. Normal sections show robust numbers of NeuN^+^ and PV^+^ cells interspersed throughout (Fig. 7B ii). No significant differences in NeuN^+^ cells were observed between EAE groups and normal groups. However, a significant decrease in PV^+^ cells was observed in middle and late EAE brain sections (F (3,30) = 6.939, middle: p = 0.0110, late: p = 0.0012) as compared to the normal group (Fig. 7B ii, v, vi).

### mRNA transcripts related to demyelination, inflammation, and neuronal damage are upregulated in late EAE

We next searched for potential molecular changes in the optic nerves of chronic EAE mice to gain some insight into potential changes during established MS disease. Changes in total brain mRNA expression were determined utilizing a Nanostring neuropathology gene expression panel. The “Neuropathology panel” contains 770 genes that provide unique cell profiling data for measuring the abundance of neurons, astrocytes, microglia, OLs, and endothelial cells (Supplementary Table 2). Past studies have shown significant changes in associated genes in the CNS during EAE^51–53^.

**Fig. 8:**
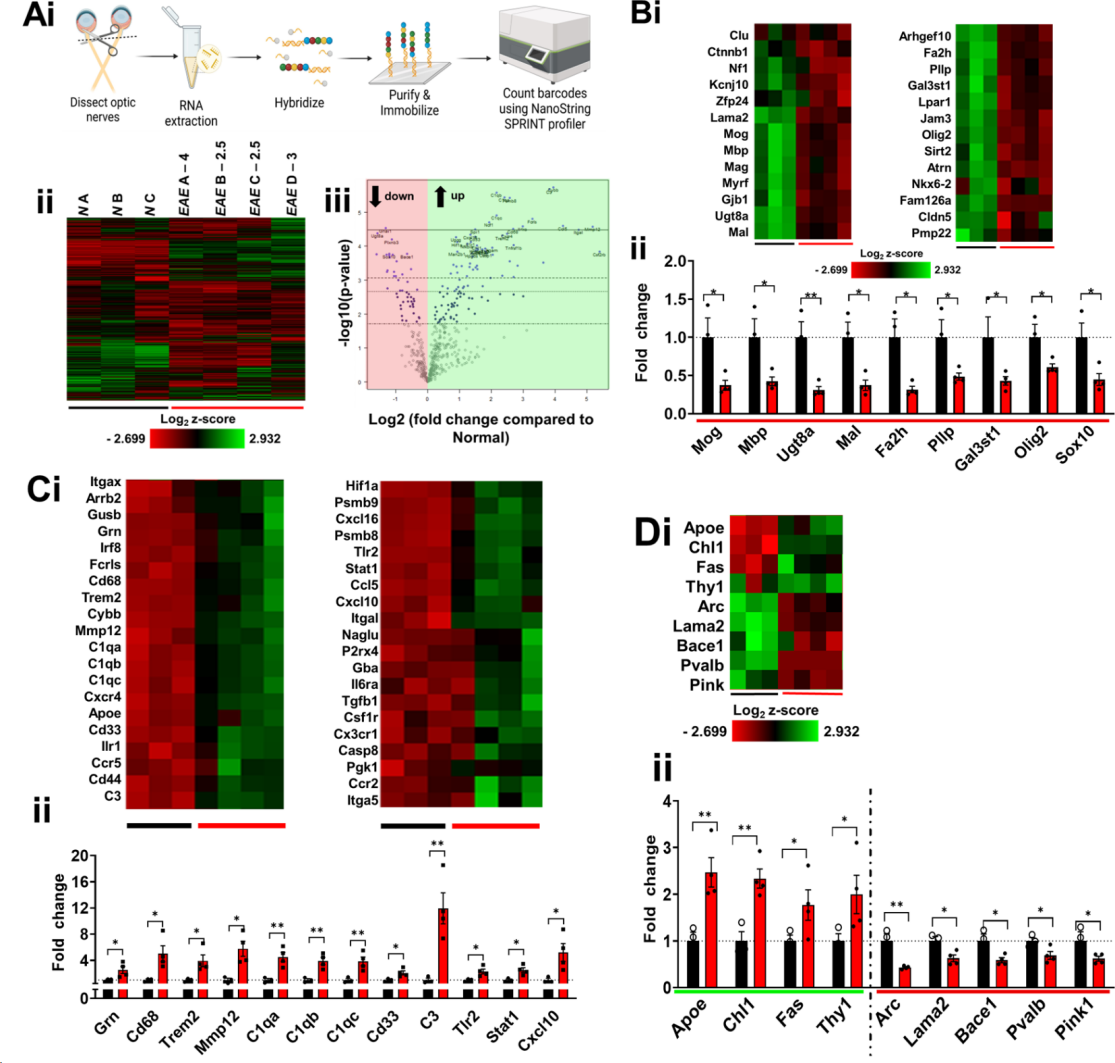
(**A**). To assess the differences in gene transcripts during late EAE, NanoString gene analysis using the Neuropathology panel on normal and late EAE optic nerves (**A i**). C57BL/6 mice were induced with EAE and at the late EAE timepoint, optic nerves were dissected (n = 4/group). C57BL/6 with just CFA and PTX was used as a normal control (NC, n = 3). Heat map of all genes from Neuropathology panels. Gene expressions are depicted from low expression (red) to high expression (green). Heat map generated from normalized gene expression data from normal (*N* A-C) and late EAE (*EAE* A-D) optic nerves using nSolver software. Clinical EAE scores are depicted for each late sample. (**An ii**) Differential expression analysis results of late EAE compared to normal control values demonstrates genes are being upregulated and downregulated during disease. Top 25 genes most significantly upregulated were identified based on fold change (x-axis) vs. p-value (y-axis). Four adjusted p-value cutoffs in each plot are as follows from bottom (dashed line) to top (solid line): < 0.50, < 0.10, < 0.05, < 0.01. Heat map of selected myelination genes from normal and late EAE stage optic nerves using NanoString Neuropathology panel (**B i**). Fold change of late EAE samples compared to normal for myelination genes from normalized gene counts. Selected genes (*Mog, Mbp, Ugt8a, Mal, Fa2h, Pllp, Gal3st1, Olig2, and Sox10*) show significant downregulation compared to normal optic nerves (**B ii**). Heat map of selected inflammation genes from normal and late EAE stage optic nerves using (**C ii**) Fold change of late EAE samples compared to normal for inflammation genes from normalized gene counts (**C i**). Selected genes (*Grn, Cd68, Trem2, Mmp12, C1qa, C1qb, C1qc, Cd33, C3, Tlr2, Stat2, and Cxcl10*) show significant upregulation compared to normal optic nerves. **D**. Neuronal health genes are upregulated and downregulated in optic nerves from mice with late EAE disease. (**D i**) Heat map of selected neuronal health genes from normal and late EAE stage optic nerves using NanoString Neuropathology panel. (**D ii**) Fold change of late EAE samples compared to normal for neuronal health genes from normalized gene counts. Genes such as *Apoe, Chl1, Fas, and Thy1* show significant upregulation while other genes like *Arc, Lama2, Bace1, Pvalb, and Pink1* show significant downregulation compared to normal optic nerves. All graphs represent mean + SEM. *p < 0.05, **p < 0.01, ***p < 0.001, ****p < 0.0001 by one-tailed student’s t-test. Figure 8Ai created in Biorender, Feri M. (2023) Biorender.com/s83j027.

Raw data and fold change of all genes from normal (n = 3) and late EAE (n = 4) are presented in Supplementary Table 3. 598 genes were found to be decreased significantly in late EAE using predefined cut-offs for selection (p < 0.05 with a log2 fold-change in the same direction) compared to normal (Fig. 8A i, ii). Using the volcano plot to demonstrate differentially expressed genes gave an insight into upregulated and downregulated genes during late EAE (Fig. 8A iii). Genes that were maximally downregulated in EAE were related to axon myelination, *Ugt8a* (t = 3.856, df = 5, pp = 0.0060), *Fa2h* (t = 3.298, df = 5, p = 0.0108), and *Mal* (t = 3.406, df = 5, p = 0.0096). *Ugt8a* is a UDP galactosyltransferase 8A and is important for myelination and lipid metabolism and has also been shown to be downregulated in the brain of EAE animals^54^. *Fa2h* is fatty acid 2-hydroxylase that catalyzes the synthesis of 2-hydroxysphingolipids, a subset of sphingolipids that contain 2-hydroxy fatty acids, important lipids for forming myelin and it is important for CNS demyelination and remyelination^55^. *MAL* encodes for a myelin and lymphocyte protein, a regulator of the recruitment of myelin protein PLP to membrane microdomains^56^. In addition to these genes, many other myelin genes such as *Sox10* (t = 3.048, df = 5, p = 0.0143)*, Gal3st1* (t = 2.441, df = 5, p = 0.0293)*, Olig2* (t = 2.594, df = 5, p = 0.0243)*, Pllp* (t = 2.565, df = 5, p = 0.0252)*, Mbp* (t = 2.689, df = 5, p = 0.0217) and *Mog* (t = 2.785, df = 5, p = 0.0193) were significantly downregulated during late EAE *(*Fig. 8Bi, ii).

In EAE groups, the complement *C3* gene was significantly upregulated (t = 3.891, df = 5, p = 0.0058) *(*Fig. 8C i, ii) and is important for the classical, alternative, and lectin pathways of complement activation, and its cleavage products C3a and C3b mediate several functions in the context of inflammation. The upregulation of C3 inhibits axon myelination and increases axon fragmentation^57^. In addition, other key inflammation genes upregulated in late EAE are complement *C1qa* (t = 4.058, df = 5, p = 0.0049)*, C1qb* (t = 4.283, df = 5, p = 0.0039), and *C1qc* (t = 3.437, df = 5, p = 0.0092)*.* These are known to be involved in the pathogenesis of multiple neuroinflammatory and neurodegenerative conditions including MS. Increases in these genes have been previously observed in the optic nerve, spinal cord and brain^28,52^ and have been shown to be directly involved in RGC loss^33^. There are other inflammatory genes such as *Trem2* (t = 2.725, df = 5, p = 0.0208)*, Mmp12* (t = 3.464, df = 5, p = 0.0090),* Cxcl10* (*t* = *2.575, df* = *5, p* = *0,0259, Stat1* (t = 3.331, df = 5, p = 0.0104)*, Cd33* (t = 2.863, df = 5, p = 0.0176)*, and Cd68* (t = 2.775, df = 5, p = 0.0196) genes *(*Fig. 8C i, ii).

Besides demyelination and inflammation, axon damage is another component of MS and EAE pathology. Many neuronal integrity genes such as *Apoe* (t = 3.650, df = 5, p = 0.0074)*, Chl1* (t = 4.546, df = 5, p = 0.0031), *Fas* (Mann Whitney test, p = 0.0286), and *Thy1* (Mann Whitney test, p = 0.0286) are upregulated. Interestingly, apolipoprotein E (*Apoe*) is a major transporter of cholesterol and extracellular lipids in the brain, where it not only mediates cholesterol exchange between non-neuronal and neuronal cell functions, but also acts as a ligand in receptor-mediated endocytosis of lipoprotein particles and has been demonstrated to play a role in antigen presentation. During EAE, expression of ApoE is elevated within the CNS, particularly by dendritic cells, and is linked to EAE pathology^58^. In contrast, genes such as *Arc* (t = 4.255, df = 5, p = 0.0040)*, Bace1* (t = 2.938, df = 5, p = 0.0162)*, Pvalb* (t = 2.646, df = 5, p = 0.0228)*, Pink1* (t = 2.175, df = 5, p = 0.0408) and *Lama2* (t = 3.202, df = 5, p = 0.0120) are significantly downregulated during EAE (Fig. 8D i-ii).The highlighted gene changes in 8B, C, and D, name and function are detailed in Supplementary Table 4. 

## Discussion

Optic neuritis is inflammation of the optic nerve and is the first sign of MS for over 20% of MS patients^59^. A major study, the Optic Neuritis Treatment Trial (ONTT)^4,60^, has significantly improved our understanding of optic neuritis and its relationship with the onset of MS. The study established that patients with idiopathic ON have a very high risk of developing clinical MS^60^. 25% of optic neuritis patients with no MRI lesions developed MS, and 50% of optic neuritis patients with 1 or more lesions developed MS within 5 years. 47% (with 1 lesion) and 72% (more than 1 lesion) of optic neuritis patients developed MS within 15 years^4,60^. Thus, optic neuritis can predict, delay, and possibly modify the rest of MS if diagnosed early, confirmed by conventional MRI, and combined with initial treatment^59,61^. Along with optic neuritis, RNFL abnormalities due to axon loss by the appearance of abnormalities on fundoscopy was recorded^62^ but were not quantifiable until the discovery of OCT that allows the quantitative cross-sectional imaging of the RNFL. MS patients with or without optic neuritis often show pathological thinning or thickening of various retinal layers throughout disease progression, specifically, the RNFL and GCL + IPL^29,63,64^. Recent progress in the assessment of visual dysfunction due to optic neuritis and subsequent MS progression has become easier with translationally relevant methods such as MRIs, VEPs, and OCT. However, there is still a significant gap in understanding the pathological consequences of these functional correlates. A better understanding of visual dysfunction in the context with MS can be achieved by investigating functional and pathological changes longitudinally in the mouse model of MS, EAE^20,23,65^. Numerous studies have been performed using EAE as a model for optic neuritis. In addition, we and others have used optic neuritis as a model to screen various therapeutic options to mitigate inflammation, demyelination, and neurodegeneration^23,66–68^. However, it has been difficult to assess the best therapeutic window as most therapies have shown partial treatment effects. Thus, to understand the effects of early, middle, and late EAE clinical disease on visual function and pathology, we performed a longitudinal EAE study.

In the preceding work, we show significant EAE-induced decreases in the RNFL at chronic disease stages, similar to observations in MS patients, who show higher levels of RNFL atrophy with disease progression^69,70^. Unlike our present and past study^23^ and the study performed by Tassoni et al.^28^, Mey et al.^20^ showed a significant decrease in RNFL layer at middle and late EAE disease. Interestingly, all 3 studies also showed a decrease in RGCs in the middle and late EAE disease. These differences could arise due to changes in EAE induction or data analysis.

The amplitude of the electronegative A-wave and electropositive B-wave in ERG reflects photoreceptor function^71^ and inner retinal function, specifically rod-bipolar cell activity^72^. In our study, retinal pathology identified by OCT was associated with functional deficits in ERGs. A-wave amplitudes and latencies, results of photoreceptor activity, were both impaired in early EAE. ERGs obtained from MS patients show conflicting results with respect to photoreceptor response time and amplitude of response, with some studies reporting no differences compared to normal controls^73,74^, and others showing an impaired response^75,76^. The contradictory responses reported in MS may be due to the differential timing of assessments. We report impairments in the A-wave only in early EAE, and prior to onset, but not at peak disease or chronic disease. The transient nature of the response in EAE indicates that some MS studies may have been completed outside of the window of observable photoreceptor dysfunction. Because the A-wave is associated with photoreceptor activity, and the photoreceptors are the first step in visual processing, it is important to characterize early deficits in these cell types during MS and EAE. In contrast, deficits in B-wave amplitudes and latencies are consistently reported in MS patients^73–76^. These are associated with cells post-synaptic to the photoreceptors including bipolar cells, Müller glia, and RGCs^77,78^. The deficits observed in the A-wave early in EAE, and the B-wave at all EAE timepoints indicate significant pathology within the neural retina.

Demyelination was also associated with early pathological increases in VEP latencies in EAE, which were attenuated more during early EAE disease and seem to resolve during the late EAE stages. These results support deficits observed in ON in which there is acute vision loss followed by partial recovery^6^. One potential explanation for this is that axons which are partially demyelinated at early disease stages can still conduct, albeit more slowly than fully myelinated axons. Demyelination of the axon results in a conduction block^79^ and as the severity of demyelination and axon damage progresses, these axons stop contributing to the VEP and thus no longer delay VEP peak times. Robust axons which were minimally myelinated, and fire similarly to normally myelinated axons, could become primary contributors to the EAE VEP, and thus return the latency recorded to normal levels. There is a progressive decrease in VEP N2 amplitudes. Because the VEP amplitude is a compound action potential recorded in V1, it is a measure of the number of V1 neurons which can be recruited to fire in response to the flash stimulus at the retina^80^. However, the P1 amplitude of the VEP during early EAE remains unchanged compared to normal, in spite of increased latency due to significant demyelination. Potentially, many V1 neurons would still contribute to the V1 amplitude even if they were partially demyelinated. The RPE is one of the essential parts of the blood retinal barrier, regulates the motion of small molecules and restricts the passageway of cells and macromolecules from the circulation. Early EAE groups showed a transient decrease in RPE that resolved in the middle and late EAE. Further investigation is required to relate these changes of progression of EAE related retinal degeneration.

The visual pathway exhibits significant deficits in MS which include demyelinated lesions throughout white matter tracts^14^ and gray matter atrophy^7,8,43,50,81^. Significant demyelination occurred in the optic nerve and the LGN during middle and late EAE, however, significant demyelination in the optic nerve and the visual cortex axons occurred early and persisted throughout the disease. Some spontaneous recovery of visual function could be due to a reorganization of sodium channels after prolonged demyelination. Changes in sodium channel expression and redistribution have been reported in the EAE optic nerve^82^, and support the observation that demyelinated axons exhibit an abrogated but not abolished ability to conduct potentials^83^. Further investigations of sodium channel distribution in the optic tract are necessary to elucidate this potential mechanism.

The current study does not explore the contribution of immune cells to EAE-induced visual dysfunction at various stages of the disease. Iba1 immunostaining was used solely to assess microglial activation during EAE. A more detailed analysis of T cells and macrophage subtypes is necessary to fully understand the role of inflammation at different stages of EAE. Microglia and macrophages accumulate in regions undergoing active demyelination and neurodegeneration in MS and EAE. There, they may amplify dysfunctional signals from CNS or immune system cells and produce a number of pro-inflammatory factors which can exacerbate myelin loss and axon damage^84^. Increases in the number of resident astrocytes and changes to morphology and molecular expression are observed in response to CNS injury^85^. These effects are often associated with blood brain barrier compromise, production of pro-inflammatory cytokines and chemokines, enhanced neuroinflammation, and scar formation in MS and EAE^86^. Astrogliosis was observed in white matter tracts at all EAE time points. It is unclear why early demyelination was not associated with overt microglial activation in EAE, as acute demyelinating lesions in MS are generally characterized by the presence of macrophages and microglia throughout the lesion area^87^. Among other roles, these pro-inflammatory cells engulf damaged myelin and oligodendrocytes as well as normal appearing white matter from MS patients^88^ and as such play a major role in demyelination. However, an insight to how demyelination could proceed in the optic tract, in the absence of these cells could be provided by the presence of hypertrophied and activated astrocytes, which also play a major role in myelin removal under disease conditions^89^.

In the dLGN and V1, increases in microglial presence and astrogliosis were observed throughout EAE and may play a role in the observed disruption of PV^+^ dendrites in the dLGN, and PV^+^ neuron loss in V1. In fact, inflammatory demyelination has been shown to negatively impact inhibitory synapses in the dLGN and PV^+^ interneurons in the cortex^90–94^. Furthermore, PV^+^ neurons are myelinated and may be particularly susceptible to inflammatory demyelinating events^47^.

Axonal damage and loss are well-known features of MS pathophysiology and are primary indicators of disability outcomes. Damaged axons are present throughout active MS lesions and at the borders of inactive lesions^95–97^. In the current work, we show the presence of some damaged axons within the optic tract during early EAE. The majority of axons at this timepoint were intact, but a few swollen endbulbs, indicative of transection, were present during peak and late EAE. The sequence of events we show throughout EAE disease follow a Wallerian degeneration-like pattern where transection leads to axonal swelling and beading followed by fragmentation and clearance of axonal debris by surrounding glia^81,98,99^. Demyelination is seen at early EAE and appears to precede overt axonal damage at peak EAE. This is similar to reports in the optic nerve which show demyelination at early EAE and extensive axonal damage at peak EAE^100^. However, we can rule out axon damage that occurred independent of demyelination as reported in histopathological analysis of early MS lesions^97,101^. Widespread axon damage early in disease has important therapeutic implications. While most therapeutic targets have focused on reducing inflammation and promoting remyelination, current treatments are unsatisfactory in addressing axon damage. Detection of early signs of axon damage such as axon swellings during early EAE introduces the idea of early intervention with neuroprotective agents following immunomodulatory and remyelinating drugs at peak disease. Recently, pharmacological inhibitors targeting the intrinsic NADase activity of sterile alpha and TIR motif containing 1 (SARM1) show protection against a model of peripheral neuropathy^102,103^. SARM1’s role in initiating axon damage has been well studied in various mouse models of other neurodegenerative diseases. Viar et al. demonstrated that the deletion of SARM1 delays axon degeneration in early EAE but does not exhibit long-term protection later in disease^104^. These findings show different results in comparison to models of traumatic brain injury and amyotrophic lateral sclerosis where SARM1 deletion shows greater protection^105,106^. Increasing demand for effective axon damage inhibitors will increase the development and testing of new reagents to inhibit SARM1 to test in neurodegenerative diseases with extensive axon damage such as MS.

High numbers of activated microglia and macrophages were associated with higher levels of axon fragmentation in the optic nerve during middle and late EAE, indicating a possible role for pro-inflammatory microglia in precipitating axonal loss. This is similarly observed in MS lesions in which there is a correlation between the number of macrophages and the extent of axonal damage^95^. Similar results showing optic nerve axon swellings with neurofilament IHC longitudinally during EAE was observed^107^. A better understanding of the cellular pathways triggering optic nerve axon damage in EAE, as well as identification of specific molecular targets that would provide a required knowledge foundation for the development of new therapies to alleviate axon damage.

In this study, we aimed to identify key myelin, inflammation, and axon health related genes in established late EAE. Nanostring analysis of optic nerves from normal and late EAE disease revealed many elevated inflammation-related genes (Supplementary Table 2). Genes of the complement system were amongst those most upregulated in each of the studied regions. *C1qa*, *C1qb* and *C1qc* subunits of the C1q protein as well as *C3, C4a*, and *Itgam* were found to be upregulated similar to that seen in the EAE spinal cord, optic nerve, and brain^28,52^. Immune cells-related genes and chemokines, primary immune cell recruitment mediators to both lymphoid and non-lymphoid tissues *Cd68, Ccr2, Mmp12, Mmp19, Cxcl16, Cxcl10, Cx3cr1, Trem2,* and *Stat1* were upregulated in the optic nerve and were also shown to be upregulated in the spinal cord and brain from EAE animals. Interestingly, compared to ON astrocytes from normal animals, an increase in C3 expression was seen in astrocytes from EAE females but not in astrocytes of EAE males^28^. A significant reduction in myelin-related genes (e.g., *Sox2*, *Mbp*, *Mog*, *Pllp*, *Ugt8a*, *Myrf1*) was observed in EAE optic nerve. This was accompanied by an upregulation of genes involved in axon degeneration, including *Apoe*, *Chl1*, *Fas*, and *Thy1*. The activity-regulated cytoskeletal (*Arc*) gene encodes a protein that is critical for memory consolidation which is affected in both MS and EAE. The β-site amyloid precursor protein cleaving enzyme 1 (Bace1) has been associated with cognitive dysfunction, amyloid deposition and neuroinflammation and its levels in the cerebrospinal fluid of MS patients have been implicated in the severity of MS disease. Pink1 is a putative mitochondrial serine/threonine kinase, which protects cells against oxidative stress-induced apoptosis. A decrease of the *Pink1* gene expression in EAE optic nerves indicated potential EAE induced changes in mitochondria and increased axonal pathology. Pink1 levels in the CSF have been implicated in MS pathology (Cossu et al., 2021). A decrease in another subset of axon-related genes important for maintaining axon health such as, *Arc*, *Lama2*, *Bace1*, *Pvalb* was observed. These gene changes give an insight into how inflammatory demyelinating changes are simultaneously accompanied by genes that initiate neurodegeneration. In the future, a longitudinal RNA study from the optic nerve and other white matter regions in the brain will help us understand if there is a gradual or sudden onset that initiated neurodegeneration that persists through late disease.

In summary, we aimed to correlate the functional data with EAE pathology, but the complexity of linking VEP and ERG outcomes to specific structures or neuronal populations made these correlations challenging. The only significant correlation we found was a decrease in retinal ganglion cell (RGC) numbers during middle EAE, which led to a reduction in the retinal nerve fiber layer (RNFL) as observed with OCT in the late stages of EAE. Overall, the pathophysiology of the anterior visual pathway during EAE is marked by extensive demyelination early in the disease, followed by irreversible neurodegeneration in the middle and late stages. These events are associated with increased inflammation and neurodegeneration in white matter. To mitigate visual pathway pathology, early therapeutic intervention with remyelinating and immunomodulatory agents may be necessary to reduce demyelination and inflammatory responses and prevent irreversible axon and neuronal damage. If early treatment is not possible, however, the prognosis for therapeutic outcomes may be poor.

## Methods

### Ethics statement

All mouse studies and mouse procedures were conducted in accordance with ARRIVE guidelines, National Institutes of Health guidelines and approved by the Institutional Care and Use of Laboratory Animals Committee (IACUC) at the University of California, Riverside (approved animal welfare assurance #123). Mice were maintained in an Association for Assessment and Accreditation of Laboratory Animal Care-accredited facility under 12 h light/dark cycle and fed standard mouse chow.

#### Mice

C57BL/6 and B6.Cg‐Tg(Thy1‐YFP)16Jrs/J mice (JAX #003709) were backcrossed to wild‐type C57BL/6 mice for more than five generations were bred and housed at UCR vivarium facilities. Thy1-YFP transgenic mice were used to assess visual pathway axonal changes during EAE^23^. Mice were kept on a 12‐hour light/dark cycle with unrestricted access to food and water.

#### EAE induction

Eight- to 12-week-old mice were induced with active EAE using MOG_35-55_ peptide as previously described^18,23,24^. Mice were induced with EAE and approximately 10–15 mice/EAE timepoint were used for various outcomes. (Fig. 1 iii). Starting at day 7 post induction (7dpi) mice were scored for disease severity and scored daily. The clinical scoring was defined as: 0, unaffected; 1, complete tail limpness; 2, failure to right upon attempt to roll over; 3, partial hind limb paralysis; 4, complete hind limb paralysis; and 5, moribund. Animals were evaluated at three timepoints: early (~ 8–17 dpi), middle (~ 18–35 dpi), and late (~ 36–47 dpi). Four complete EAE experiments were conducted, with 30 to 40 animals used per experiment. Animals that did not develop clinical signs of EAE were excluded from the study. Three representative EAE experiments are shown in Fig. 1 ii. A subgroup of mice was kept as control mice (no MOG_35-55_ peptide) and were named the normal group. (Fig. 1).

#### Optical coherence tomography (OCT)

OCT was performed according to previously published methods^23^. OCT data plots of mouse retinas were acquired with spectral domain‐OCT (R2200 840 nm HHP; Leica, Deerfield, IL) during early, middle, and late disease as previously described and detailed in^23^. Retinal structure was analyzed lateral to the optic nerve. Automatic segmentation of retinal layers was performed with Bioptigen Diver 3.0 software (Leica Microsystems, Deerfield, IL). In brief, animals were anesthetized and maintained with 10% ketamine and xylazine as previously published. One thousand A-scans and one hundred B-scans were taken to generate each OCT image. Each image was taken 3 times and averaged. Software segmented individual retinal images avoided the inclusion of blood vessel diameter specifically during RNFL calculations.

#### Electroretinograms (ERG) and visual evoked potentials (VEP)

ERG and VEPs were measured during early, middle, and late EAE disease using OcuScience handheld multi‐species electroretinography (Henderson, NV) similar to as previously described (Sekyi et al., 2021). Briefly, animals were dark adapted (5 h) followed by continuous anesthesia with 2% isoflurane during acquisition. Stainless steel subdermal electrodes (F-Needle Electrode (F-E2); OcuScience, Henderson, NV) were inserted at the base of the tail (ground electrode), and on either side of the snout (reference electrodes). ERG recordings were taken at flashes: 5 each 2S (0.50 Hz), intensity: 3000mcd/m^2^, data: 30mS – flash – 300mS, and background 0 mcd/m^2^. VEP recordings were taken at 71 each 2S (0.50 Hz), intensity: 3000mcd/m^2^, data: 30mS – flash – 300mS, and background 0 mcd/m^2^. Mice were averaged with a minimum of 5 responses for ERGs and 25 responses for VEPs. Traces were filtered for 60 Hz noise and a low-pass filter of 150 Hz with MATLAB. Trace baselines were adjusted to 0 at the onset of the light stimulus. VEP responses were smoothed (3rd order Savitzky–Golay, 50 points per window) (43). MATLAB was used to identify and measure ERG and VEP peak amplitudes and latencies.

#### Perfusions, tissue preparation, and Immunohistochemistry (IHC)

Mice were deeply anesthetized with isoflurane and intracardially perfused with ice‐cold PBS followed by 10% formalin (Fisher Scientific, Hampton, NH). Eyes, optic nerves, and brain were collected, cryoprotected, sectioned, and subjected to IHC as previously described^23^.

#### Microscopy, quantification, and statistics

40 µm thick retina, optic nerve, and brain sections were subjected to IHC and imaged using an Olympus BX61 spinning disk confocal microscope at 10 × and 40 × magnifications. Z‐stack images were acquired, and projection images were compiled using Slidebook 6 and cellSens software (Intelligent Imaging Innovations Inc, Santa Monica, CA). Antibodies used are listed in Supplementary Table 1. Immunofluorescence intensity and cell numbers in regions of interest were quantified using NIH ImageJ software (v1. 50i http://rsb.info.nih.gov/ij/). To assess the status of RGCs during EAE progression, IHC was performed on horizontal retinal sections. A grid was laid on 40X image of the retina. Optic nerve, and brain sections containing optic tract, dLGN, and VC. Clearly immunostained markers for RGCs, OLs were counted in the same magnification and same area. Immunostaining of astrocytes, immune cells, myelin intensity and others were represented as % area. % Areas were assessed by splitting the RGB image colors and converting these individual color images (representing antibody staining) to grayscale. The staining intensity threshold was set after making sure the images background was equally adjusted across the images. The region of interest i.e., optic tract, dLGN, and VC were delineated as shown in (Sekyi et al., 2021), followed by quantification of the staining intensity percent in that region using inbuilt macros in ImageJ. Results were analyzed in GraphPad Prism for statistical significance.

#### RNA extraction

A modified method was used to isolate RNA from a single optic nerve. Normal and EAE-induced (late timepoint) C57BL/6 mice were decapitated, and optic nerves were isolated in under 3 min. After isolating the optic nerve, 100 µl of cold RIPA buffer was added and frozen temporarily at -20 °C. Zirconium Oxide microbeads were added to each tube and sample homogenized using a Bullet Blender set at a speed of “9” for 5 min. RLT Plus solution from Qiagen RNeasy Plus Micro Kit (Qiagen, Hilden, Germany) was added and the mixture re-homogenized two more times. Immediately after, RNA isolation was performed using the Qiagen RNeasy Plus Micro Kit according to manufacturer’s protocol. RNA concentration and purity was determined using a Nanodrop spectrophotometer (RRID:SCR_016517), then samples were analyzed using Agilent 2100 Bioanalyzer/Advanced Analytics Fragment Analyzer (RRID:SCR_018043). RIN values above 7 were adequate for Nanostring profiling.

*NanoString nCounter Gene Expression Assay* Optic nerve RNA (150µL/50 ng of RNA per sample) from individual mice were used to perform nCounter gene expression assays (NanoString Technologies, Seattle, WA) using the nCounter Mouse Neuropathology panel (770 genes) according to manufacturer’s instructions. Briefly, 50 ng of unamplified RNA was hybridized with the reporter code set at 65 °C for 18 h. Samples were spun down, nuclease free water was added to the samples, and then the samples were loaded into the nCounter cartridge. The cartridge was run on the Nanostring nCounter SPRINT Profiler (RRID:SCR_021712). Data was exported and analyzed using Nanostring nSolver and Advanced Analysis Software (RRID:SCR_021712). Normalized linear counts for all genes in the panel were used in fold change analysis of control and late EAE genes.

#### Statistics

For EAE clinical scores, ordinary two-way ANOVA with Bonferroni multiple comparisons test was performed to determine differences between normal and EAE animals. For IHC, three sections per mouse were taken for each area of interest in the brain. For retina and optic nerve tissues, three sections were taken per mouse. There were 8 mice per EAE timepoint. We used Bonferroni post hoc test as it is more conservative and reliable. IHC data were analyzed using ordinary one-way ANOVA with Bonferroni’s multiple comparisons test. For ERG, VEP, and OCT studies both eyes were assessed. Data were analyzed using student’s *t*-test or ordinary one-way ANOVA with Bonferroni’s multiple comparisons test. For nCounter gene expression analysis, normalized counts were used to calculate Log_2_ fold changes. Statistical analysis was performed in GraphPad Prism 10 software using one-tailed student’s *t*-test. Differences were considered significant at the *p < 0.05, **p < 0.01, ***p < 0.001, ****p < 0.0001 level.

## Electronic supplementary material

Below is the link to the electronic supplementary material.

Supplementary Material 1

Supplementary Material 2

Supplementary Material 3

## Data Availability

Raw and fold change gene data has been submitted as Supplementary figures.

## Abbreviations

EAE: Experimental autoimmune encephalomyelitis
MS: Multiple sclerosis
ON: Optic neuritis

## References

[1] Solomon, A. J. & Corboy, J. R. The tension between early diagnosis and misdiagnosis of multiple sclerosis. *Nat. Rev. Neurol.* **13**, 567. 10.1038/nrneurol.2017.106 (2017). 28799551 10.1038/nrneurol.2017.106

[2] Poser, C. M. & Brinar, V. V. Diagnostic criteria for multiple sclerosis: An historical review. *Clin. Neurol. Neurosurg.* **106**, 147–158. 10.1016/j.clineuro.2004.02.004 (2004). 10.1016/j.clineuro.2004.02.004 15177763

[3] McDonald, W. I. et al. Recommended diagnostic criteria for multiple sclerosis: Guidelines from the International Panel on the diagnosis of multiple sclerosis. *Ann. Neurol.* **50**, 121–127 (2001). 11456302 10.1002/ana.1032

[4] Volpe, N. J. The optic neuritis treatment trial: A definitive answer and profound impact with unexpected results. *Arch Ophthalmol.* **126**, 996–999. 10.1001/archopht.126.7.996 (2008). 18625952 10.1001/archopht.126.7.996

[5] Buzzard, T. Atrophy of the optic nerve as a symptom of chronic disease of the central nervous system. *Br. Med. J.* **2**, 770–784 (1893).

[6] Toosy, A. T., Mason, D. F. & Miller, D. H. Optic neuritis. *Lancet Neurol.* **13**, 83–99. 10.1016/S1474-4422(13)70259-X (2014). 24331795 10.1016/S1474-4422(13)70259-X

[7] Balk, L. J. et al. Bidirectional trans-synaptic axonal degeneration in the visual pathway in multiple sclerosis. *J. Neurol. Neurosurg. Psychiatry* **86**, 419–424. 10.1136/jnnp-2014-308189 (2015). 24973342 10.1136/jnnp-2014-308189

[8] Gabilondo, I. et al. Trans-synaptic axonal degeneration in the visual pathway in multiple sclerosis. *Ann. Neurol.* **75**, 98–107. 10.1002/ana.24030 (2014). 24114885 10.1002/ana.24030

[9] Bock, M. et al. Impairment of contrast visual acuity as a functional correlate of retinal nerve fibre layer thinning and total macular volume reduction in multiple sclerosis. *Br. J. Ophthalmol.* **96**, 62–67. 10.1136/bjo.2010.193581 (2012). 21378002 10.1136/bjo.2010.193581

[10] Villoslada, P., Cuneo, A., Gelfand, J., Hauser, S. L. & Green, A. Color vision is strongly associated with retinal thinning in multiple sclerosis. *Mult. Scler.* **18**, 991–999. 10.1177/1352458511431972 (2012). 22291035 10.1177/1352458511431972

[11] Fisher, J. B. et al. Relation of visual function to retinal nerve fiber layer thickness in multiple sclerosis. *Ophthalmology* **113**, 324–332. 10.1016/j.ophtha.2005.10.040 (2006). 16406539 10.1016/j.ophtha.2005.10.040

[12] Backner, Y. & Levin, N. Keep your eyes wide open: On visual- and vision-related measurements to better understand multiple sclerosis pathophysiology. *J. Neuroophthalmol.* **38**, 85–90. 10.1097/wno.0000000000000634 (2018). 29438265 10.1097/WNO.0000000000000634

[13] Kuchling, J., Brandt, A. U., Paul, F. & Scheel, M. Diffusion tensor imaging for multilevel assessment of the visual pathway: Possibilities for personalized outcome prediction in autoimmune disorders of the central nervous system. *EPMA J.* **8**, 279–294. 10.1007/s13167-017-0102-x (2017). 29021839 10.1007/s13167-017-0102-x PMC5607151

[14] Graham, S. L. & Klistorner, A. Afferent visual pathways in multiple sclerosis: A review. *Clin. Exp. Ophthalmol.* **45**, 62–72. 10.1111/ceo.12751 (2017). 27011293 10.1111/ceo.12751

[15] Monsalve, P. et al. Retinal ganglion cell function in recovered optic neuritis: Faster is not better. *Clin. Neurophysiol.* **129**, 1813–1818. 10.1016/j.clinph.2018.06.012 (2018). 29981956 10.1016/j.clinph.2018.06.012

[16] Balcer, L. J. et al. Validity of low-contrast letter acuity as a visual performance outcome measure for multiple sclerosis. *Mult. Scler.* **23**, 734–747. 10.1177/1352458517690822 (2017). 28206829 10.1177/1352458517690822 PMC5407511

[17] Mangiardi, M. et al. An animal model of cortical and callosal pathology in multiple sclerosis. *Brain Pathol.* **21**, 263–278. 10.1111/j.1750-3639.2010.00444.x (2011). 21029240 10.1111/j.1750-3639.2010.00444.x PMC5233755

[18] Hasselmann, J. P., Karim, H., Khalaj, A. J., Ghosh, S. & Tiwari-Woodruff, S. K. Consistent induction of chronic experimental autoimmune encephalomyelitis in C57BL/6 mice for the longitudinal study of pathology and repair. *J. Neurosci. Methods.*10.1016/j.jneumeth.2017.04.003 (2017). 10.1016/j.jneumeth.2017.04.003 PMC5749979 28396177

[19] Liu, M., Duggan, J., Salt, T. E. & Cordeiro, M. F. Dendritic changes in visual pathways in glaucoma and other neurodegenerative conditions. *Exp. Eye Res.* **92**, 244–250. 10.1016/j.exer.2011.01.014 (2011). 21310146 10.1016/j.exer.2011.01.014

[20] Mey, G. M. et al. Visual imaging as a predictor of neurodegeneration in experimental autoimmune demyelination and multiple sclerosis. *Acta Neuropathol. Commun.* **10**, 87. 10.1186/s40478-022-01391-y (2022). 35706005 10.1186/s40478-022-01391-y PMC9199245

[21] Marenna, S. et al. Functional evolution of visual involvement in experimental autoimmune encephalomyelitis. *Mult. Scler. J. Exp. Transl. Clin.* **6**, 2055217320963474. 10.1177/2055217320963474 (2020). 35145730 10.1177/2055217320963474 PMC8822451

[22] Jin, J. et al. Glial pathology and retinal neurotoxicity in the anterior visual pathway in experimental autoimmune encephalomyelitis. *Acta Neuropathol. Commun.* **7**, 125. 10.1186/s40478-019-0767-6 (2019). 31366377 10.1186/s40478-019-0767-6 PMC6670238

[23] Sekyi, M. T. et al. Alleviation of extensive visual pathway dysfunction by a remyelinating drug in a chronic mouse model of multiple sclerosis. *Brain Pathol.* **31**, 312–332. 10.1111/bpa.12930 (2021). 33368801 10.1111/bpa.12930 PMC8018057

[24] Karim, H. et al. Increase in chemokine CXCL1 by ERbeta ligand treatment is a key mediator in promoting axon myelination. *Proc. Natl. Acad. Sci. U.S.A.*10.1073/pnas.1721732115 (2018). 10.1073/pnas.1721732115 PMC6004485 29844175

[25] Karim, H. et al. Analogues of ERbeta ligand chloroindazole exert immunomodulatory and remyelinating effects in a mouse model of multiple sclerosis. *Sci. Rep.* **9**, 503. 10.1038/s41598-018-37420-x (2019). 30679747 10.1038/s41598-018-37420-x PMC6345788

[26] Sergott, R. C. Optical coherence tomography: Measuring in-vivo axonal survival and neuroprotection in multiple sclerosis and optic neuritis. *Curr. Opin. Ophthalmol.* **16**, 346–350 (2005). 16264344 10.1097/01.icu.0000188705.67815.0e

[27] Pulicken, M. et al. Optical coherence tomography and disease subtype in multiple sclerosis. *Neurology* **69**, 2085–2092. 10.1212/01.wnl.0000294876.49861.dc (2007). 18040015 10.1212/01.wnl.0000294876.49861.dc

[28] Tassoni, A. et al. The astrocyte transcriptome in EAE optic neuritis shows complement activation and reveals a sex difference in astrocytic C3 expression. *Sci. Rep.* **9**, 10010. 10.1038/s41598-019-46232-6 (2019). 31292459 10.1038/s41598-019-46232-6 PMC6620300

[29] Al-Louzi, O. A. et al. Outer retinal changes following acute optic neuritis. *Mult. Scler.* **22**, 362–372. 10.1177/1352458515590646 (2016). 26209589 10.1177/1352458515590646 PMC4724567

[30] Walter, S. D. et al. Ganglion cell loss in relation to visual disability in multiple sclerosis. *Ophthalmology* **119**, 1250–1257. 10.1016/j.ophtha.2011.11.032 (2012). 22365058 10.1016/j.ophtha.2011.11.032 PMC3631566

[31] Pinto, L. H., Invergo, B., Shimomura, K., Takahashi, J. S. & Troy, J. B. Interpretation of the mouse electroretinogram. *Doc Ophthalmol* **115**, 127–136. 10.1007/s10633-007-9064-y (2007). 17636411 10.1007/s10633-007-9064-y PMC3786689

[32] Green, A. J., McQuaid, S., Hauser, S. L., Allen, I. V. & Lyness, R. Ocular pathology in multiple sclerosis: Retinal atrophy and inflammation irrespective of disease duration. *Brain* **133**, 1591–1601. 10.1093/brain/awq080 (2010). 20410146 10.1093/brain/awq080 PMC2877904

[33] Gharagozloo, M. et al. Complement component 3 from astrocytes mediates retinal ganglion cell loss during neuroinflammation. *Acta Neuropathol.* **142**, 899–915. 10.1007/s00401-021-02366-4 (2021). 34487221 10.1007/s00401-021-02366-4 PMC8713426

[34] Qian, Z. et al. Longitudinal in vivo evaluation of retinal ganglion cell complex layer and dendrites in mice with experimental autoimmune encephalomyelitis. *Exp. Eye Res.* **237**, 109708. 10.1016/j.exer.2023.109708 (2023). 37913917 10.1016/j.exer.2023.109708

[35] Esiri, M. M. & Reading, M. C. Macrophage populations associated with multiple sclerosis plaques. *Neuropathol. Appl. Neurobiol.* **13**, 451–465. 10.1111/j.1365-2990.1987.tb00074.x (1987). 3328828 10.1111/j.1365-2990.1987.tb00074.x

[36] Sun, S. W., Liang, H. F., Schmidt, R. E., Cross, A. H. & Song, S. K. Selective vulnerability of cerebral white matter in a murine model of multiple sclerosis detected using diffusion tensor imaging. *Neurobiol. Dis.* **28**, 30–38. 10.1016/j.nbd.2007.06.011 (2007). 17683944 10.1016/j.nbd.2007.06.011 PMC2905808

[37] Singh, S. et al. Relationship of acute axonal damage, Wallerian degeneration, and clinical disability in multiple sclerosis. *J. Neuroinflamm.* **14**, 57. 10.1186/s12974-017-0831-8 (2017). 10.1186/s12974-017-0831-8 PMC5356322 28302146

[38] Budde, M. D., Xie, M., Cross, A. H. & Song, S. K. Axial diffusivity is the primary correlate of axonal injury in the experimental autoimmune encephalomyelitis spinal cord: A quantitative pixelwise analysis. *J. Neurosci.* **29**, 2805–2813. 10.1523/JNEUROSCI.4605-08.2009 (2009). 19261876 10.1523/JNEUROSCI.4605-08.2009 PMC2673458

[39] Pisa, M. et al. Anterior optic pathway pathology in CNS demyelinating diseases. *Brain* **145**, 4308–4319. 10.1093/brain/awac030 (2022). 35134111 10.1093/brain/awac030 PMC9762948

[40] Kerschensteiner, D. & Guido, W. Organization of the dorsal lateral geniculate nucleus in the mouse. *Visual Neurosci.* **34**, E008–E008. 10.1017/S0952523817000062 (2017). 10.1017/S0952523817000062 PMC6380502 28965501

[41] Rompani, S. B. et al. Different modes of visual integration in the lateral geniculate nucleus revealed by single-cell-initiated transsynaptic tracing. *Neuron* **93**, 767-776.e766. 10.1016/j.neuron.2017.01.028 (2017). 28231464 10.1016/j.neuron.2017.01.028 PMC5330803

[42] Corbett, J. J. & Chen, J. In *Fundamental Neuroscience for Basic and Clinical Applications (Fifth Edition)* (eds Duane E. Haines & Gregory A. Mihailoff) 286–305.e281 (Elsevier, 2018).

[43] Papadopoulou, A. et al. Damage of the lateral geniculate nucleus in MS: Assessing the missing node of the visual pathway. *Neurology* **92**, e2240–e2249. 10.1212/wnl.0000000000007450 (2019). 30971483 10.1212/WNL.0000000000007450 PMC6537126

[44] Watson, C. *Visual System*. 783–795 (Elsevier, 2012).

[45] Zonouzi, M. et al. Individual Oligodendrocytes Show Bias for Inhibitory Axons in the Neocortex. *Cell Rep.* **27**, 2799-2808 e2793. 10.1016/j.celrep.2019.05.018 (2019). 31167127 10.1016/j.celrep.2019.05.018 PMC7968376

[46] Tomassy, G. S. et al. Distinct profiles of myelin distribution along single axons of pyramidal neurons in the neocortex. *Science* **344**, 319–324. 10.1126/science.1249766 (2014). 24744380 10.1126/science.1249766 PMC4122120

[47] Micheva, K. D. *et al.* A large fraction of neocortical myelin ensheathes axons of local inhibitory neurons. *Elife* **5**, 10.7554/eLife.15784 (2016). 10.7554/eLife.15784 PMC4972537 27383052

[48] Jukkola, P., Guerrero, T., Gray, V. & Gu, C. Astrocytes differentially respond to inflammatory autoimmune insults and imbalances of neural activity. *Acta Neuropathol. Commun.* **1**, 70–70. 10.1186/2051-5960-1-70 (2013). 24252623 10.1186/2051-5960-1-70 PMC3893391

[49] Eilam, R. et al. Astrocyte disruption of neurovascular communication is linked to cortical damage in an animal model of multiple sclerosis. *Glia* **66**, 1098–1117. 10.1002/glia.23304 (2018). 29424049 10.1002/glia.23304

[50] Gabilondo, I. et al. The influence of posterior visual pathway damage on visual information processing speed in multiple sclerosis. *Mult. Scler.* **23**, 1276–1288. 10.1177/1352458516676642 (2017). 28273763 10.1177/1352458516676642

[51] Cardona, S. M. et al. Role of the Fractalkine receptor in CNS autoimmune inflammation: New approach utilizing a mouse model expressing the human CX3CR1(I249/M280) variant. *Front. Cell. Neurosci.* **12**, 365. 10.3389/fncel.2018.00365 (2018). 30386211 10.3389/fncel.2018.00365 PMC6199958

[52] Becquart, P., Vilarino-Guell, C. & Quandt, J. A. Enhanced expression of complement and microglial-specific genes prior to clinical progression in the MOG-experimental autoimmune encephalomyelitis model of multiple sclerosis. *Brain Res. Bull.* **165**, 63–69. 10.1016/j.brainresbull.2020.09.010 (2020). 32979467 10.1016/j.brainresbull.2020.09.010

[53] Hendrickx, D. A. E. et al. Gene expression profiling of multiple sclerosis pathology identifies early patterns of demyelination surrounding chronic active lesions. *Front. Immunol.* **8**, 1810. 10.3389/fimmu.2017.01810 (2017). 29312322 10.3389/fimmu.2017.01810 PMC5742619

[54] Omura, S. et al. Bioinformatics analyses determined the distinct CNS and peripheral surrogate biomarker candidates between two mouse models for progressive multiple sclerosis. *Front. Immunol.* **10**, 516. 10.3389/fimmu.2019.00516 (2019). 30941144 10.3389/fimmu.2019.00516 PMC6434997

[55] Dimas, P. *et al.* CNS myelination and remyelination depend on fatty acid synthesis by oligodendrocytes. *Elife* **8**, 10.7554/eLife.44702 (2019). 10.7554/eLife.44702 PMC6504237 31063129

[56] Bijlard, M. et al. MAL is a regulator of the recruitment of myelin protein PLP to membrane microdomains. *PLoS One* **11**, e0155317. 10.1371/journal.pone.0155317 (2016). 27171274 10.1371/journal.pone.0155317 PMC4865042

[57] Peterson, S. L., Nguyen, H. X., Mendez, O. A. & Anderson, A. J. Complement protein C3 suppresses axon growth and promotes neuron loss. *Sci. Rep.* **7**, 12904. 10.1038/s41598-017-11410-x (2017). 29018286 10.1038/s41598-017-11410-x PMC5635131

[58] Shin, S. et al. Apolipoprotein E mediation of neuro-inflammation in a murine model of multiple sclerosis. *J. Neuroimmunol.* **271**, 8–17. 10.1016/j.jneuroim.2014.03.010 (2014). 24794230 10.1016/j.jneuroim.2014.03.010 PMC4042395

[59] Phuljhele, S., Kedar, S. & Saxena, R. Approach to optic neuritis: An update. *Indian J. Ophthalmol.* **69**, 2266–2276. 10.4103/ijo.IJO_3415_20 (2021). 34427197 10.4103/ijo.IJO_3415_20 PMC8544067

[60] Rizzo, J. F. 3rd. & Lessell, S. Risk of developing multiple sclerosis after uncomplicated optic neuritis: A long-term prospective study. *Neurology* **38**, 185–190 (1988). 3340278 10.1212/wnl.38.2.185

[61] Beck, R. W. et al. High- and low-risk profiles for the development of multiple sclerosis within 10 years after optic neuritis: Experience of the optic neuritis treatment trial. *Arch. Ophthalmol.* **121**, 944–949. 10.1001/archopht.121.7.944 (2003). 12860795 10.1001/archopht.121.7.944

[62] Hoyt, W. F. Fundoscopic changes in the retinal nerve-fibre layer in chronic and acute optic neuropathies. *Trans. Ophthalmol. Soc. U.K.* **1962**(96), 368–371 (1976). 1071914

[63] Gabilondo, I. et al. Dynamics of retinal injury after acute optic neuritis. *Ann. Neurol.* **77**, 517–528. 10.1002/ana.24351 (2015). 25559267 10.1002/ana.24351

[64] Saidha, S. et al. Visual dysfunction in multiple sclerosis correlates better with optical coherence tomography derived estimates of macular ganglion cell layer thickness than peripapillary retinal nerve fiber layer thickness. *Mult. Scler. J.* **17**, 1449–1463. 10.1177/1352458511418630 (2011). 10.1177/1352458511418630 21865411

[65] Redler, Y. & Levy, M. Rodent models of optic neuritis. *Front. Neurol.* **11**, 580951. 10.3389/fneur.2020.580951 (2020). 33224094 10.3389/fneur.2020.580951 PMC7669908

[66] Liu, P. et al. Differential effects of SARM1 inhibition in traumatic glaucoma and EAE optic neuropathies. *Mol. Ther. Nucl. Acids* **32**, 13–27. 10.1016/j.omtn.2023.02.029 (2023). 10.1016/j.omtn.2023.02.029 PMC10025007 36950280

[67] Anders, J. J., Elwood, B. W., Kardon, R. H. & Gramlich, O. W. Acriflavine, a HIF-1 inhibitor, preserves vision in an experimental autoimmune encephalomyelitis model of optic neuritis. *Front. Immunol.* **14**, 1271118. 10.3389/fimmu.2023.1271118 (2023). 37942317 10.3389/fimmu.2023.1271118 PMC10628762

[68] Poon, M. M. et al. Targeting the muscarinic M1 receptor with a selective, brain-penetrant antagonist to promote remyelination in multiple sclerosis. *Proc. Natl. Acad. Sci. U.S.A.* **121**, e2407974121. 10.1073/pnas.2407974121 (2024). 39083422 10.1073/pnas.2407974121 PMC11317586

[69] Behbehani, R. et al. Optical coherence tomography segmentation analysis in relapsing remitting versus progressive multiple sclerosis. *PLoS One* **12**, e0172120. 10.1371/journal.pone.0172120 (2017). 28192539 10.1371/journal.pone.0172120 PMC5305239

[70] Garcia-Martin, E. et al. Retinal and optic nerve degeneration in patients with multiple sclerosis followed up for 5 years. *Ophthalmology* **124**, 688–696. 10.1016/j.ophtha.2017.01.005 (2017). 28187977 10.1016/j.ophtha.2017.01.005

[71] Penn, R. D. & Hagins, W. A. Signal transmission along retinal rods and the origin of the electroretinographic a-wave. *Nature* **223**, 201–204. 10.1038/223201a0 (1969). 4307228 10.1038/223201a0

[72] Robson, J. G., Maeda, H., Saszik, S. M. & Frishman, L. J. In vivo studies of signaling in rod pathways of the mouse using the electroretinogram. *Vis. Res.* **44**, 3253–3268. 10.1016/j.visres.2004.09.002 (2004). 15535993 10.1016/j.visres.2004.09.002

[73] Papakostopoulos, D., Fotiou, F., Hart, J. C. & Banerji, N. K. The electroretinogram in multiple sclerosis and demyelinating optic neuritis. *Electroencephalogr. Clin. Neurophysiol.* **74**, 1–10 (1989). 2463143 10.1016/0168-5597(89)90045-2

[74] Hamurcu, M., Orhan, G., Saricaoglu, M. S., Mungan, S. & Duru, Z. Analysis of multiple sclerosis patients with electrophysiological and structural tests. *Int. Ophthalmol.* **37**, 649–653. 10.1007/s10792-016-0324-2 (2017). 27538913 10.1007/s10792-016-0324-2

[75] Hanson, J. V. M. et al. Outer retinal dysfunction in the absence of structural abnormalities in multiple sclerosis. *Invest. Ophthalmol. Vis. Sci.* **59**, 549–560. 10.1167/iovs.17-22821 (2018). 10.1167/iovs.17-22821 29372255

[76] Forooghian, F. et al. Electroretinographic abnormalities in multiple sclerosis: Possible role for retinal autoantibodies. *Doc. Ophthalmol.* **113**, 123–132. 10.1007/s10633-006-9022-0 (2006). 16972082 10.1007/s10633-006-9022-0

[77] Perlman, I. In *Webvision: The Organization of the Retina and Visual System* (eds H. Kolb, E. Fernandez, & R. Nelson) (University of Utah Health Sciences Center Copyright: (c) 2019 Webvision., 1995). 21413389

[78] Smith, B. J., Wang, X., Chauhan, B. C., Cote, P. D. & Tremblay, F. Contribution of retinal ganglion cells to the mouse electroretinogram. *Doc Ophthalmol* **128**, 155–168. 10.1007/s10633-014-9433-2 (2014). 24659322 10.1007/s10633-014-9433-2

[79] Smith, M. E. Phagocytosis of myelin in demyelinative disease: A review. *Neurochem. Res.* **24**, 261–268 (1999). 9972873 10.1023/a:1022566121967

[80] Creel, D. J. In *Webvision: The Organization of the Retina and Visual System* (eds H. Kolb, E. Fernandez, & R. Nelson) (University of Utah Health Sciences Center Copyright: (c) 2019, Webvision., 1995). 21413389

[81] Shen, T. et al. Differing structural and functional patterns of optic nerve damage in multiple sclerosis and neuromyelitis optica spectrum disorder. *Ophthalmology* **126**, 445–453. 10.1016/j.ophtha.2018.06.022 (2019). 30060979 10.1016/j.ophtha.2018.06.022

[82] Craner, M. J., Lo, A. C., Black, J. A. & Waxman, S. G. Abnormal sodium channel distribution in optic nerve axons in a model of inflammatory demyelination. *Brain* **126**, 1552–1561. 10.1093/brain/awg153 (2003). 12805113 10.1093/brain/awg153

[83] Bostock, H. & Sears, T. A. The internodal axon membrane: Electrical excitability and continuous conduction in segmental demyelination. *J Physiol* **280**, 273–301. 10.1113/jphysiol.1978.sp012384 (1978). 690876 10.1113/jphysiol.1978.sp012384 PMC1282659

[84] Carson, M. J. Microglia as liaisons between the immune and central nervous systems: Functional implications for multiple sclerosis. *Glia* **40**, 218–231. 10.1002/glia.10145 (2002). 12379909 10.1002/glia.10145 PMC2693029

[85] Sofroniew, M. V. Molecular dissection of reactive astrogliosis and glial scar formation. *Trends Neurosci.* **32**, 638–647. 10.1016/j.tins.2009.08.002 (2009). 19782411 10.1016/j.tins.2009.08.002 PMC2787735

[86] Lopes-Pinheiro, M. A. et al. Immune cell trafficking across the barriers of the central nervous system in multiple sclerosis and stroke. *Biochim. Biophys. Acta (BBA) Mol. Basis Dis.* **1862**, 461–471. 10.1016/j.bbadis.2015.10.018 (2016). 10.1016/j.bbadis.2015.10.018 26527183

[87] Kuhlmann, T. et al. An updated histological classification system for multiple sclerosis lesions. *Acta Neuropathol.* **133**, 13–24. 10.1007/s00401-016-1653-y (2017). 27988845 10.1007/s00401-016-1653-y

[88] Hendrickx, D. A., Schuurman, K. G., van Draanen, M., Hamann, J. & Huitinga, I. Enhanced uptake of multiple sclerosis-derived myelin by THP-1 macrophages and primary human microglia. *J. Neuroinflamm.* **11**, 64. 10.1186/1742-2094-11-64 (2014). 10.1186/1742-2094-11-64 PMC4108133 24684721

[89] Morizawa, Y. M. et al. Reactive astrocytes function as phagocytes after brain ischemia via ABCA1-mediated pathway. *Nat. Commun.* **8**, 28. 10.1038/s41467-017-00037-1 (2017). 28642575 10.1038/s41467-017-00037-1 PMC5481424

[90] Lapato, A. S. et al. Chronic demyelination-induced seizures. *Neuroscience* **346**, 409–422. 10.1016/j.neuroscience.2017.01.035 (2017). 28153692 10.1016/j.neuroscience.2017.01.035 PMC5394933

[91] Clements, R. J., McDonough, J. & Freeman, E. J. Distribution of parvalbumin and calretinin immunoreactive interneurons in motor cortex from multiple sclerosis post-mortem tissue. *Exp. Brain Res.* **187**, 459–465. 10.1007/s00221-008-1317-9 (2008). 18297277 10.1007/s00221-008-1317-9

[92] Potter, L. E. et al. Altered excitatory-inhibitory balance within somatosensory cortex is associated with enhanced plasticity and pain sensitivity in a mouse model of multiple sclerosis. *J. Neuroinflamm.* **13**, 142. 10.1186/s12974-016-0609-4 (2016). 10.1186/s12974-016-0609-4 PMC4901403 27282914

[93] Falco, A., Pennucci, R., Brambilla, E. & de Curtis, I. Reduction in parvalbumin-positive interneurons and inhibitory input in the cortex of mice with experimental autoimmune encephalomyelitis. *Exp. Brain Res.* **232**, 2439–2449. 10.1007/s00221-014-3944-7 (2014). 24770856 10.1007/s00221-014-3944-7 PMC4055863

[94] Araujo, S. E. S. et al. Inflammatory demyelination alters subcortical visual circuits. *J. Neuroinflamm.* **14**, 162. 10.1186/s12974-017-0936-0 (2017). 10.1186/s12974-017-0936-0 PMC5562979 28821276

[95] Ferguson, B., Matyszak, M. K., Esiri, M. M. & Perry, V. H. Axonal damage in acute multiple sclerosis lesions. *Brain* **120**(Pt 3), 393–399 (1997). 9126051 10.1093/brain/120.3.393

[96] McDonald, W. I., Perry, V. H. & Anthony, D. C. Axon damage and repair in multiple sclerosis. *Philos. Trans. R. Soc. Lond. Ser. B Biol. Sci.* **354**, 1641–1647. 10.1098/rstb.1999.0509 (1999). 10603617 10.1098/rstb.1999.0509 PMC1692675

[97] Trapp, B. D. et al. Axonal transection in the lesions of multiple sclerosis. *N. Engl. J. Med.* **338**, 278–285 (1998). 9445407 10.1056/NEJM199801293380502

[98] Adalbert, R. et al. Intra-axonal calcium changes after axotomy in wild-type and slow Wallerian degeneration axons. *Neuroscience* **225**, 44–54. 10.1016/j.neuroscience.2012.08.056 (2012). 22960623 10.1016/j.neuroscience.2012.08.056

[99] Yang, J. et al. Regulation of axon degeneration after injury and in development by the endogenous calpain inhibitor calpastatin. *Neuron* **80**, 1175–1189. 10.1016/j.neuron.2013.08.034 (2013). 24210906 10.1016/j.neuron.2013.08.034

[100] Fairless, R. et al. Preclinical retinal neurodegeneration in a model of multiple sclerosis. *J. Neurosci.* **32**, 5585–5597. 10.1523/JNEUROSCI.5705-11.2012 (2012). 22514320 10.1523/JNEUROSCI.5705-11.2012 PMC6703474

[101] Kornek, B. et al. Multiple sclerosis and chronic autoimmune encephalomyelitis: A comparative quantitative study of axonal injury in active, inactive, and remyelinated lesions. *Am. J. Pathol.* **157**, 267–276 (2000). 10880396 10.1016/S0002-9440(10)64537-3 PMC1850217

[102] Hughes, R. O. et al. Small molecule SARM1 inhibitors recapitulate the SARM1(-/-) phenotype and allow recovery of a metastable pool of axons fated to degenerate. *Cell Rep.* **34**, 108588. 10.1016/j.celrep.2020.108588 (2021). 33406435 10.1016/j.celrep.2020.108588 PMC8179325

[103] Bosanac, T. et al. Pharmacological SARM1 inhibition protects axon structure and function in paclitaxel-induced peripheral neuropathy. *Brain* **144**, 3226–3238. 10.1093/brain/awab184 (2021). 33964142 10.1093/brain/awab184 PMC8634121

[104] Viar, K., Njoku, D., Secor McVoy, J. & Oh, U. *Sarm1* knockout protects against early but not late axonal degeneration in experimental allergic encephalomyelitis. *PLOS ONE* (2020). 10.1371/journal.pone.0235110 PMC7316289 32584865

[105] Marion, C. M., McDaniel, D. P. & Armstrong, R. C. Sarm1 deletion reduces axon damage, demyelination, and white matter atrophy after experimental traumatic brain injury. *Exp Neurol* **321**, 113040. 10.1016/j.expneurol.2019.113040 (2019). 31445042 10.1016/j.expneurol.2019.113040

[106] White, M. A. et al. Sarm1 deletion suppresses TDP-43-linked motor neuron degeneration and cortical spine loss. *Acta Neuropathol. Commun.* **7**, 166. 10.1186/s40478-019-0800-9 (2019). 31661035 10.1186/s40478-019-0800-9 PMC6819591

[107] Drake, S. S. *et al.* 3-dimensional immunostaining and automated deep-learning based analysis of nerve degeneration. *Int. J. Mol. Sci.* **23**, 10.3390/ijms232314811 (2022). 10.3390/ijms232314811 PMC9739543 36499143

