# Supplementary material for: Demyelination and neurodegeneration early in experimental autoimmune encephalomyelitis contribute to functional deficits in the anterior visual pathway
Source: Sci Rep. 2024 Oct 14;14:24048. doi: 10.1038/s41598-024-73792-z (PMC11473523; doi:10.1038/s41598-024-73792-z)
Supplement: Supplementary file 3 — Supplementary Material 3 [file 41598_2024_73792_MOESM3_ESM.pdf]

Title: Demyelination and Neurodegeneration early in experimental autoimmune encephalomyelitis contribute to functional deficits in the anterior visual pathway.

Author names and affiliations: Maria T. Sekyi[1#], Micah Feri[1#], Shane Desfor[1], Kelley C. Atkinson[1], Batis Golestany[1], Fernando Beltran[1], and Seema K. Tiwari-Woodruff[1*]

Supplemental Material

Supplementary Table 1:

Antibodies used for immunohistochemistry

**TABLE 1**			
**Antibody**	**Target**	**Vendor**	**Catalog #**
MBP	Myelin basic protein	Abcam	ab40390
NF200	Neurofilament	Sigma	N4142
NeuN	Neuronal Nuclear antigen	Invitrogen	MA5-33103
CD86	Cluster of Differentiation 86	Cell Signaling	E2G8P
C1Q	Complement component 1q	Abcam	4.8
CD45	Cluster of differentiation 45;	Abcam	EPR28934-536
GFAP	Glial fibrillary acidic protein	Invitrogen	180063
Iba-1	Ionized calcium binding adaptor molecule 1	Wako	019-19741
PV	Parvalbumin (clone PARV-19)	EMD Millipore	MAB1572
RBPMS	RNA binding protein with multiple splicing	PhosphoSolutions	1830

Supplementary Table 2:
Neuropathology panel, Nanostring (attached)

Supplementary Table 3:
Raw data and fold change of genes from isolated optic nerves of normal (n=3) and EAE (n=4) groups of mice (attached)

Supplementary Table 4:
Names and Function of Key Genes that were modified.

A Myelin Related Genes

Gene	Name	Function
**Mog**	Myelin oligodendrocyte glycoprotein	Completion and maintenance of the myelin sheath and cell-cell communication
**Mbp**	Myelin basic protein	Major constituent of the myelin sheath
**Ugt8a**	UDP galactosyltransferase 8A	Catalyzes a key enzymatic step in biosynthesis of galactocerebrosides which are abundant in the myelin membrane.
**Mal**	Myelin and lymphocyte protein	Plays a role in the formation, stabilization and maintenance of glycosphingolipid-rich membrane domains
**Fa2h**	fatty acid 2-hydroxylase	Catalyzes a step that produces 2-hydroxy fatty acids, which are building blocks of sphingolipids and glycosphingolipids in neural tissue
**Pllp**	Plasmolipin	Involved in myelination, located in compact myelin and membrane raft
**Gal3st1**	Galactose-3-O-sulfotransferase 1	Encodes galactosylceramide sulfotransferase, which catalyzes the sulfation of membrane glycolipids including the final step in the synthesis of sulfatide, a major lipid component of the myelin sheath.
**Olig2**	Oligodendrocyte transcription factor 2	Needed for oligodendrocyte and motor neuron differentiation
**Sox10**	SRY-Box Transcription Factor 10	oligodendrocyte maturation

B Inflammation Related Genes

Gene	Name	Function
**Grn**	granulin	key regulator of lysosomal function and as a growth factor involved in inflammation, wound healing and cell proliferation
**CD68**	Cluster of differentiation 68	Highly expressed in cells of monocyte lineage, circulating macrophages, and tissue macrophages. Also plays a role in phagocytic activies of tissue macrophages
**Trem2**	Triggering Receptor Expressed On Myeloid Cells 2	a membrane protein that forms a receptor signaling complex with the TYRO protein tyrosine kinase binding protein. Functions in the immune response and may be involved in chronic inflammation by triggering the production of constitutive inflammatory cytokines
**Mmp12**	Matrix Metallopeptidase 12	involved in tissue remodeling, wound repair, progression of atherosclerosis and tumor invasion.
**C1qa**	Complement C1q subcomponent subunit A	A-chain polypeptide of serum complement subcomponent C1q to form first component of complement system
**C1qb**	Complement C1q subcomponent subunit B	B-chain polypeptide of serum complement subcomponent C1q to form first component of complement system
**C1qc**	Complement C1q subcomponent subunit C	C-chain polypeptide of serum complement subcomponent C1q to form first component of complement system
**Cd33**	CD33 molecule	Enables protein phosphatase binding activity and sialic acid binding activity. Involved in several processes, including negative regulation of cytokine production; negative regulation of monocyte activation; and positive regulation of protein tyrosine phosphatase activity.
**C3**	Complement C3	Activation of the complement system

**Tlr2**	Toll-like receptor 2	Forms heterodimers with TLR2 and TLR6 as the initial step in the events that lead to innate immune responses, development of adaptive immunity, and protection from immune sequelae related to infection
**Stat1**	Signal transducer and activator of trascription 1	Plays a key role in the immune response against viruses and other pathogens by initiating signalin from type I, type II, and type III IFNs.
**Cxcl10**	C-X-C motif chemokine ligand 10	binds to CXCR3 to mediate immune responses through the activation and recruitment of leukocytes such as T cells, eosinophils, monocytes and NK cells. In oligodendrocytes, it inhibits OPC proliferation and myelination.

C Neurodegeneration related genes

Gene	Name	Function
**Apoe**	Apolipoprotein E	apolipoprotein, a protein associating with lipid particles, that mainly functions in lipoprotein-mediated lipid transport between organs via the plasma and interstitial fluids
**Chl1**	cell adhesion molecule L1-like	Extracellular matrix and cell adhesion protein that plays a role in nervous system development and in synaptic plasticity
**Fas**	Fas receptor	Encodes for a death receptor on the surface of cells that leads to apoptosis if it binds to its ligand Fas ligand
**Thy1**	thymus cell antigen 1, theta	involved in cell adhesion and cell communication in cells of the immune and nervous systems
**Arc**	Activity-regulated cytoskeleton-associated protein	a key regulator of synaptic plasticity: required for protein synthesis-dependent forms of long-term potentiation (LTP) and depression (LTD) and for the formation of long-term memory
**Lama2**	laminin, alpha 2	provides instructions for making a part (subunit) of certain members of a protein family called laminins
**Bace1**	Beta-secretase 1	A transmembrane protease that catalyzes the first step in the formation of amyloid beta peptide from amyloid precursor protein.
**Pvalb**	Parvalbumin	Encodes a high affinity calcium ion-binding protein that is involved in relaxation after contraction. Present in GABAergic interneurons that are fast-spiking.
**Pink1**	PTEN induced kinase 1	encodes a serine/threonine protein kinase that localizes to mitochondria. It is thought to protect cells from stress-induced mitochondrial dysfunction
